# Nuclear preservation in the cartilage of the Jehol dinosaur *Caudipteryx*

**DOI:** 10.1038/s42003-021-02627-8

**Published:** 2021-09-24

**Authors:** Xiaoting Zheng, Alida M. Bailleul, Zhiheng Li, Xiaoli Wang, Zhonghe Zhou

**Affiliations:** 1 Institute of Geology and Paleontology, Linyi University, Linyi City, Shandong 276005 China; 2 Shandong Tianyu Museum of Nature, Pingyi, Shandong 273300 China; 3 Key Laboratory of Vertebrate Evolution and Human Origins, Institute of Vertebrate Paleontology and Paleoanthropology, 142 Xizhimenwai dajie, Beijing, 100044 China; 4 CAS Center for Excellence in Life and Paleoenvironment, Beijing, 100044 China

**Keywords:** Molecular biology, Evolution

## Abstract

Previous findings on dinosaur cartilage material from the Late Cretaceous of Montana suggested that cartilage is a vertebrate tissue with unique characteristics that favor nuclear preservation. Here, we analyze additional dinosaur cartilage in *Caudipteryx* (STM4-3) from the Early Cretaceous Jehol biota of Northeast China. The cartilage fragment is highly diagenetically altered when observed in ground-sections but shows exquisite preservation after demineralization. It reveals transparent, alumino-silicified chondrocytes and brown, ironized chondrocytes. The histochemical stain Hematoxylin and Eosin (that stains the nucleus and cytoplasm in extant cells) was applied to both the demineralized cartilage of *Caudipteryx* and that of a chicken. The two specimens reacted identically, and one dinosaur chondrocyte revealed a nucleus with fossilized threads of chromatin. This is the second example of fossilized chromatin threads in a vertebrate material. These data show that some of the original nuclear biochemistry is preserved in this dinosaur cartilage material and further support the hypothesis that cartilage is very prone to nuclear fossilization and a perfect candidate to further understand DNA preservation in deep time.

## Abstract (web summary)

Zheng et al. report on the presence of nuclear preservation in the femoral cartilage of a specimen of Caudipteryx, a theropod dinosaur. They identify the presence of chromatin threads for only the second time in any vertebrate fossil.

## Introduction

The preservation of cell nuclei in long extinct organisms is currently considered rare and exceptional. Due to the fragility of nucleic acids, nuclei are thought to degrade extremely rapidly after death (sometimes within hours *postmortem*), leaving almost no chance for these structures to enter the fossil record (e.g., ref. ^1^). However, the paleontological literature is full of histological reports of fossil tissues with exquisitely preserved nuclei and even sub-nuclear structures like nucleoli or chromosomes in multiple stages of cell division^2^. These examples are numerous and include nuclei from permafrost-preserved Cenozoic mammals, Mesozoic dinosaurs, various Cenozoic, Mesozoic, and Paleozoic plants, and even embryo-like fossil cell clusters that are more than 600 Million years (My) old (e.g., refs. ^3–7^). Recent taphonomy experiments on plants and algae have shown that nuclei are surprisingly stable and that nuclear decay is much slower than originally thought in some conditions^8,9^.

One of the finest levels of nuclear preservation is exemplified by fossilized mitotic chromosomes in permineralized plants^3,4,7,10,11^. In living organisms (Eukaryotes and Archaea), chromosomes represent the highest level of condensation of chromatin, which is composed of condensed DNA molecules coiled around histone proteins (e.g., ref. ^12^). Recently, chromosome-like chromatin threads were reported for the first time in a vertebrate fossil in the cartilage of a 75 My old duck-billed dinosaur (*Hypacrosaurus stebingeri*^6^). In this material, nuclear and cellular preservation was exquisite and never seen before in a vertebrate. Due to a few unique tissue characteristics of calcified cartilage, such as the absence of vascularization/porosity, a high mineral:organic ratio and low amounts of oxygen, it was hypothesized that this calcified tissue is very prone to cellular and nuclear preservation, most likely more so than bone^6^.

Here, we further investigate cellular and nuclear preservation in additional dinosaur cartilage material using a piece of femoral articular cartilage from a specimen of the oviraptorosaurid *Caudipteryx* (STM4-3) from the Early Cretaceous Jehol Biota. The Jehol ecosystem of Northeast China belongs to a Konservat-Lagerstätte in which most unearthed vertebrates have articulated skeletons, often accompanied by exquisite soft tissue and organ preservation^13,14^. It is logical to assume that, at least in some Jehol fossils, this morphological preservation extends to the cellular and intracellular level, including in fossilized cartilage. This tissue has already been investigated in three specimens of birds from the Jehol (i.e., in the skull of *Yanornis* and the postcrania of two different *Confuciusornis* specimens^15–18^), but in these cases, the cartilage was either not analyzed at the intracellular level^15–17^ or it did not show any obvious nuclear preservation^18^.

To investigate chondrocyte preservation in *Caudipteryx*, we used an array of microscopy methods that complement each other, including ground-sections, scanning electron microscopy (SEM), energy-dispersive X-ray spectroscopy (EDS), histochemical staining, and transmission electron microscopy (TEM). In STM4-3, we report another example of exquisitely preserved dinosaur cartilage cells, with one cell showing a nucleus with intracellular chromatin threads that have retained some of their original chemistry. We discuss the implications of our findings in the context of our understanding of nuclear fossilization in cartilage and of tissue preservation in the Jehol biota. We also discuss the importance of chemical and molecular research on cartilage cell nuclei to further understand DNA preservation in deep time.

## Results

### Specimen

**Fig. 1:**
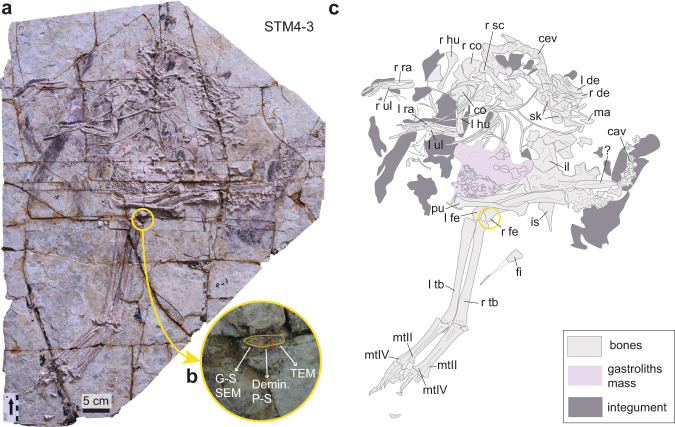
Photograph and line drawing of *Caudipteryx* STM4-3. **a** Photograph of the full slab. **b** Close-up on the right femur showing the extracted fragment outlined in yellow. **c** Line drawing of the specimen showing bones in light gray, the mass of gastroliths inside the body in pale gray and integument imprints in dark gray. cav, caudal vertebrae; cev, cervical vertebrae; Demin. P-S, demineralization and paraffin sections; fi, fibula; G-S SEM, Ground-sections and scanning electron microscopy; il, ilium; is, ischium; l co, left coracoid; l de, left dentary; l fe, left femur; l hu, left humerus; l ra, left radius; l tb, left tibiotarsus; l ul, left ulna; ma, maxilla; mtII, metatarsal II; mtIV, metatarsal IV; pu, pubis; r co, right coracoid; r de, right dentary; r fe, right femur; r hu, right humerus; r ra, right radius; r sc, right scapula; r tb, right tibiotarsus; r ul, right ulna; sk, skull elements; TEM, transmission electron microscopy.

**Fig. 2:**
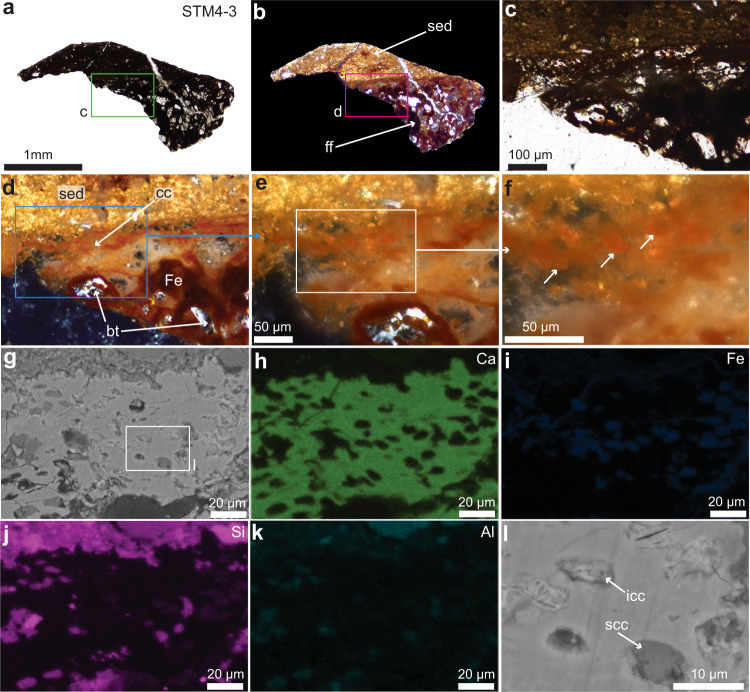
Photographs of a fragment of the articular cartilage of STM4-3 seen with multiple microscopy methods. **a** Femur fragment (ff) seen under transmitted light. **b** Fragment seen under the polarized light. **c** Close-up of the fragment (green box in **a**) showing dark tissues and overlying sediment. **d** Close-up of the fragment (pink box in **b**) seen under overexposed polarized light, showing sediments (sed), calcified cartilage (cc), and a few subchondral bone trabeculae (bt) that are birefringent. The two tissues are infiltrated with an orange to dark red material mostly composed of iron (Fe). **e** A close-up on the cartilage reveals that a lot of the chondrocyte lacunae are filled with the orange iron material. **f** Close-up of the white box in e, with arrows pointing at a few chondrocyte lacunae. **g** SEM image of approximately the same area shown in e, showing calcified cartilage with chondrocyte lacunae filled with 2 types of material: a dark gray material and a lighter material. **h**–**k** EDS analysis on this same image (**g**) showing the calcium (Ca) of the extracellular matrix, a group of chondrocyte lacunae filled with Silicon (Si) and Aluminum (Al), and another group with lacunae that are filled with iron (Fe). **l** Close-up of the white box in (**g**) showing ironized cartilage cells (icc) and silicified cartilage cells (scc).

**Fig. 3:**
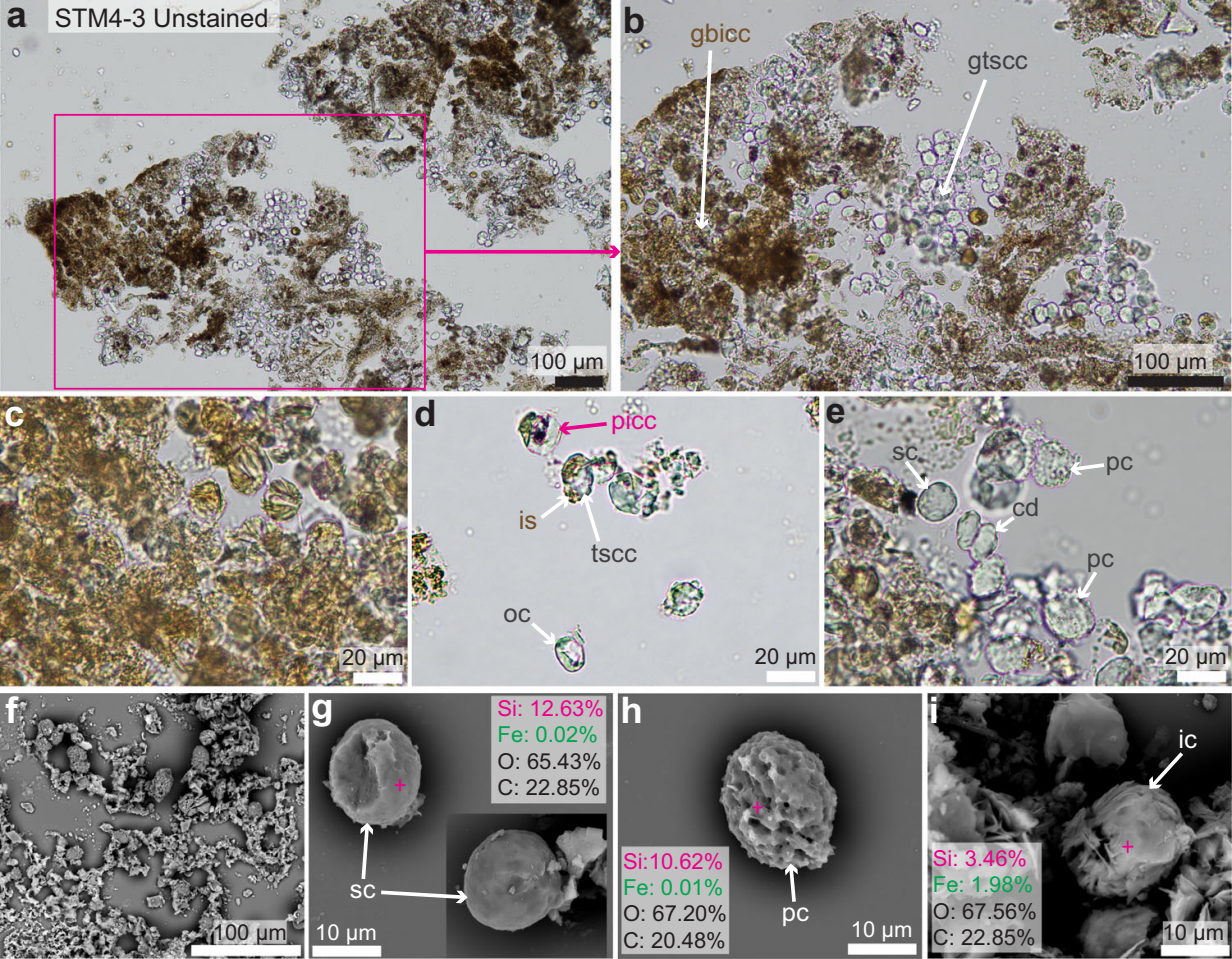
Photographs of unstained paraffin sections and SEM images of the demineralized cartilage of STM4-3. **a** One example of a 10 µm thick paraffin section of unstained, demineralized cartilage shows a dark brown material overall. **b** A close-up on this material shows that it is made of numerous globular structures representing fossilized chondrocytes. Some of them are transparent, others are brown. They represent alumino-silicified cells and ironized cells respectively. **c** Close-up on brown, ironized cartilage cells showing some striations. **d** Close-up on some ironized cells showing an iron sheath (is) that is peeling off. Other cells are apparently open (oc, open cell) and some show potential intracellular content (picc). **e** Close-up on transparent silicified cells showing two types of cells (smooth cells, sc; and porous cells, pc). **f** SEM image of a slide of demineralized cartilage sprayed with gold. **g** SEM image of two smooth silicified cells paired with an EDS analysis at the pink cross. **h** SEM-EDS image of a porous silicified cell. **i** SEM-EDS image of a striated ironized cell. Additional abbreviations: cd, cell doublet; gbicc, group of brown ironized cartilage cells; gtscc, group of transparent silicified cartilage cells; tscc, transparent silicified cartilage cell.

**Fig. 4:**
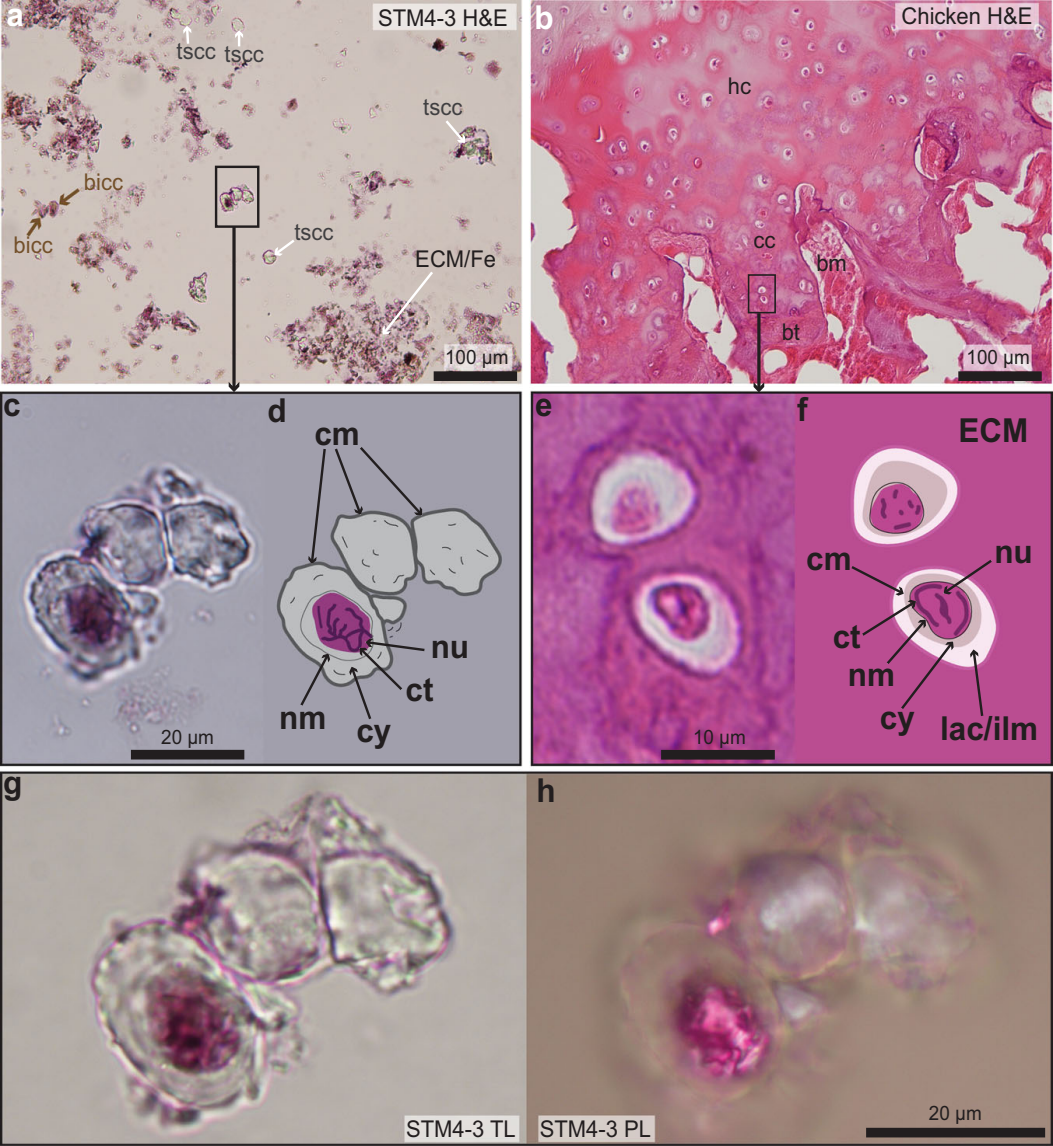
Photographs of H&E stained paraffin sections of the cartilage of STM4-3 and that of an extant chicken. **a** A slide of STM4-3 cartilage (5 µm thick) stained with H&E. Few extracellular matrix (ECM) and few chondrocytes are seen. **b** A slide of adult chicken cartilage (5 µm thick) stained with the same method and shown at the same scale for comparison with *Caudipteryx*. **c** A close-up on three *Caudipteryx* cells shows one cartilage cell with stained intracellular structures and chromatin threads in dark purple. **d** Line drawing of (**c**) showing different fossilized cellular structures, like the unstained cytoplasm (cy), the unstained nuclear membrane (nm), and the stained nucleus (nu). **e** A close-up on two chicken cells. **f** Accompanying line drawing of (**e**) showing similar cellular structures sharing the same staining pattern as the *Caudipteryx* cell. Because the chicken cells are still within their ECM, their lacunae (lac) with potential intralacunar matrix (ilm) can be seen. **g** High-magnification photograph (at ×100 with immersion oil) on the three *Caudipteryx* cells seen under transmitted light (TL). **h** Corresponding image taken under the polarized light (PL). Abbreviations: bicc, brown ironized cartilage cell; bm, bone marrow; bt, bone trabeculae; cc, calcified cartilage; cm, cell membrane; ct, chromatin thread/chromocenter; ECM/Fe, extracellular matrix/Iron; hc, hyaline cartilage; tscc, transparent silicified cartilage cell.

STM 4-3 (Shandong Tianyu Museum of Nature) is a well-preserved, complete and partially articulated specimen of *Caudipteryx*, with a femur length of about 15 cm (Fig. 1a). It also preserves the outline of original feathers and integument in a few areas, as well as a white mass of gastroliths inside its abdomical cavity (Fig. 1a, c). This specimen was collected in the Yixian Formation near Chaoyang City, Dapingfang Town in West Liaoning. One main fragment was extracted from the distal articular surface of the right femur and then further divided into three smaller fragments (Fig. 1b) for paleohistology and SEM-EDS analyses (Fig. 2), decalcified paraffin histology (Figs. 3, 4), and TEM analyses.

### Paleohistology and SEM-EDS

Under transmitted light, ground-sections of the first fragment show an extremely dark material even though the slides are relatively thin (30 µm; Fig. 2a; Supplementary Fig. 1). At higher magnification, tissues are still not easily identifiable (Fig. 2c) and only an overexposed polarized light setting allows a better identification of the preserved structures (Fig. 2b, d–f). The sample is composed of sediments covering the articular end of the femur fragment, with a thin layer of calcified cartilage laying on top of subchondral bone (Fig. 2d–f). Both the calcified cartilage and the subchondral bone are highly diagenetically altered and invaded by a red-brown material (Fig. 2d–f; Supplementary Fig. 1). At low resolution, it has the shape of a web-like network infiltrating the spaces between bony trabeculae. In the calcified cartilage (which is avascular and lacks porous spaces), this network appears to be directly percolating the cartilaginous extracellular matrix (ECM) and the chondrocyte lacunae (Fig. 2d–f). Even at high magnification under the polarized light, the chondrocyte lacunae are not easily identifiable (Fig. 2f) and individual lacunae are best observed under the SEM (Fig. 2g, l). EDS analyses showed that the red-brown network is made mostly of iron (Supplementary Note 1). When EDS is focused on the calcified cartilage at high resolution (Fig. 2g–k; Supplementary Note 2), two different types of material can be seen inside the cell lacunae: a material mostly made of iron (Fig. 2i), and a material mostly made of silicon, with lower amounts of aluminum (Fig. 2j-k). A high-resolution SEM image shows that the two types of intralacunar material also have different morphologies (Fig. 2l). A piece of well-preserved muscle was found in a second ground-section of the same fragment. It is alumino-silicified but lacks iron (Supplementary Fig. 1; Supplementary Note 3). This piece of muscle is highly birefringent under the polarized light (Supplementary Fig. 1).

### Histology and chemistry of decalcified cartilage

The second extracted fragment (Fig. 1b) was decalcified for three weeks in ethylenediaminetetraacetic acid (EDTA) and transformed into 10 µm-thick, unstained paraffin sections (Fig. 3a–e). Some dark brown areas are visible and most likely represent a combination of the iron network mixed with remnants of decalcified cartilage ECM (Fig. 3a). At higher magnification, numerous fossilized round cartilage cells can be seen (Fig. 3b). Even though the histological preservation of the calcified cartilage looked very poor in the ground-section and the cartilage cell lacunae looked dark and irregularly-shaped under transmitted light (Fig. 2), getting rid of most of the ECM by decalcification revealed in fact an exquisite cellular preservation (Fig. 3). Groups of brown, striated cells (Fig. 3b, c) and groups of transparent cells can be seen (Fig. 3b, d, e). A close look at the transparent cells reveals two different types of morphologies: cells with a smooth surface (“sc” in Fig. 3e) and cells with pores (“pc” in Fig. 3e). Based on the results from the previous slide (Fig. 2), the most logical conclusion to make based on color is that the striated, brown cells are ironized and the two types of transparent cells are alumino-silicified. However, the brown cells are transparent internally (and only covered by an external brown sheath). This can be seen in  a few cells showing an external sheath that has been peeled off (Fig. 3d). Additionally, a few cells show an open cell membrane (lowest cell in Fig. 3d) and others potentially show some intracellular content (pink arrow in Fig. 3d).

An SEM and EDS analysis performed on these decalcified cells in an adjacent paraffin slide (Fig. 3f) also showed these three different types of cellular morphologies (Fig. 3g–i) and confirmed our previous chemical interpretation based on cell color. This specimen of *Caudipteryx* indeed has transparent, smooth, and porous cells that are silicified (Fig. 3g, h; Supplementary Notes 4–5) and brown, striated cells that are ironized (Fig. 3i; Supplementary Notes 6). While it is clear that the ironized cellular sheaths are a product of diagenesis, it is unclear how and when some of the silicified cells acquired pores. However, because cellular membranes become porous during the process of cell death, the porous silicified cells may represents cells that were in the process of dying at the time of mineralization. 

### Histochemistry on decalcified cartilage

To further investigate the presence of potential intracellular and nuclear material in these cartilage cells, we stained some of the paraffin slides (this time, with a standard thickness of 5 µm) with the common histochemical stain Hematoxylin and Eosin (H&E) used worldwide in biology laboratories (Fig. 4a). Hematoxylin reveals nuclei and nucleic acids in blue/purple and Eosin stains proteins of the cytoplasm and of the ECM in varying degrees of pink^19^. For a comparison with an extant analogue, a piece of decalcified articular cartilage and subchondral bone from the limb bone of an extant adult chicken (Fig. 4b; specimen used in ref. ^20^) was stained with the same method (while avoiding possibilities of contamination; Supplementary Note 7). After staining, the chicken cartilage showed a pink ECM with chondrocyte nuclei staining purple (Fig. 4b, e). The chicken chondrocytes are naturally smaller than those in *Caudipteryx* because of the size difference between these two species, and because the cartilage cells of *Caudipteryx* additionally appear to be hypertrophic (Fig. 4a, c) unlike those in this adult chicken (Fig. 4b, e).

In the H&E stained slides of *Caudipteryx*, most of the ECM and cells were lost during the cutting and/or the staining process, but a few cells are still visible (Fig. 4a). Most silicified cartilage cells were unaffected by the H&E solution and remained transparent after staining (Fig. 4a). However, one transparent cell with an open cellular membrane showed some elongated, dark purple threads encased into a larger purple circular structure (Fig. 4c, d). This structure is clearly delimited and internal to the cell, and the area surrounding it remained unstained.

When this stained dinosaur cell is compared to stained avian chondrocytes, an identical staining pattern can be seen. The extant cells show an unstained cytoplasm located around a purple nucleus that has darker intranuclear areas (Fig. 4e, f). An unstained cytoplasm after H&E staining is not uncommon in extant material (and this can be due to a number of variables and steps in the H&E protocol^21^); and the darker intranuclear areas represent chromocenters, which are areas where chromatin is more condensed^22^. Based on the size, the morphology, the location of the structures seen in the dinosaur cell and based on the H&E staining pattern of the avian cartilage cells, the most logical conclusion is that this *Caudipteryx* cell preserves an original dinosaur nucleus (Fig. 4c). It sits within the cell cytoplasm, is delimited by a nuclear membrane and further contains darker stained material showing the morphological characteristics of condensed chromatin threads (Fig. 4c, d).

It is important to use different types of microscopes, light settings and different planes of the section to observe fine intracellular structures in fossilized cells (e.g., see ref. ^6^). At higher magnification (×100 with immersion oil), the cell nucleus still shows dark purple threads of chromatin in a paler nucleoplasm (Fig. 4g; even though the threads have a slightly different arrangement in this plane when compared to that shown in Fig. 4c). Under the polarized light, the outline of these threads is even clearer and the stained nucleus is slightly birefringent (Fig. 4h).

The last fragment was prepared for TEM sectioning (Fig. 1b) to further analyze cartilage cells and potentially identify additional nuclei at much higher resolution (Supplementary Fig. 2). However, due to the minuscule thickness of the cuts (70 nm) and the difficulty to specifically target the cartilage, we were only able to observe two bone cells from the subchondral bone with this method. These two cells were ironized, showing a sheath of iron oxides around their cell membrane, but they did not show any apparent intracellular content (Supplementary Fig. 2).

## Discussion

Cartilage is a skeletal tissue that is less studied than bone or teeth in most branches of vertebrate paleontology. In the field of forensics it has been understudied for decades as well, but recent taphonomy experiments showed that hyaline (i.e., uncalcified) articular cartilage within the joints of limb bones in mammalian carcasses is one the most durable and decay resistant soft tissues of the body (e.g., refs. ^23–25^). This can be explained by multiple factors: articular cartilage is shielded from external contaminating microbes by the surrounding tissues of the joint capsule and by subchondral bone^24,25^. It also lacks vascularization and innervation, which offers protection against microbial invasions and the alteration of organic components, most likely similar to avascular eggshells which were recently shown to offer better biomolecule preservation^26^ as well as better a DNA recovery than vascularized bone^27^. Moreover, cartilage has a low cell density and its cells have an anaerobic metabolism (e.g., refs. ^23–25^). To our knowledge and according to histology textbooks (e.g., ref. ^28^), there are only two main tissues in the vertebrate body that have cells with an anaerobic metabolism: cartilage and avascular epithelia. Chondrocytes receive nutrition and oxygen via diffusion through the avascular cartilaginous matrix and are unlike the cells of vascularized tissues which receive their oxygen directly from blood vessels (e.g., ref. ^29^). This makes chondrocytes resistant to oxygen starvation and acidosis and all of these characteristics apparently play a role in delaying the autolytic processes (i.e., the autodestruction of cells by their lytic enzymes) that should otherwise start almost immediately after death^25,30–32^. For example, chondrocytes in human articular cartilage can remain viable for at least two weeks *postmortem* at ambient temperatures^33^. In a preliminary taphonomy experiment performed on avian cartilage decaying in water, many chondrocytes in the cartilage still showed a comparable morphology to those of ‘healthy’ chondrocytes after 15 days *postmortem*^18,34^. This shows that autolysis was also apparently completely inhibited for at least 2 weeks after death in this extant avian cartilage^18,34^.

It was recently proposed that a delay or a complete inhibition of autolysis is one of the most important factors (and a pre-requisite) for the fossilization of nuclei and finer intranuclear structures in deep time^2^. Forensic studies that noted the resistance of chondrocytes mostly focus on uncalcified hyaline cartilage (or at least, they do not specifically make the distinction between decay rates in hyaline versus calcified cartilage), but it can be logically assumed that the chondrocytes embedded in a calcified ECM are even more decay resistant than hyaline cartilage cells, being further protected from external contaminants by their mineralized matrix. All of these characteristics show that it is not that surprising to observe exceptional cellular and nuclear preservation in fossilized calcified cartilage, regardless of the age of the fossil. Since chondrocyte autolysis can take a few weeks to start after death^33,34^, hypothetically neither burial nor permineralization need to be immediate for nuclear preservation to be observed in fossil cartilage. This was exemplified in the dinosaur *Hypacrosaurus*, where exceptional nuclear preservation was seen in some chondrocytes, yet the taphonomy of the nesting ground from which it was collected suggested burial was not particularly rapid^6^. This was also seen in a specimen of *Confuciusornis*^18^. In this specimen, permineralization of the tissues was extensively delayed *postmortem*, yet chondrocyte preservation (although poor) was still observed^18^.

In the Late Cretaceous *Hypacrosaurus*, two nucleated cells linked by an intercellular bridge at the end of mitosis and one dying cell with internal metaphase-like chromatin threads were observed in a ground-section^6^. Some isolated cells also reacted with the DNA fluorescent dyes propidium iodide and DAPI^6^. Here, using paraffin histology paired with the histochemical stain H&E, we report an additional example of preserved chromatin threads in chondrocytes from even older dinosaur material, from the Early Cretaceous Jehol biota. This gives further support to the previous hypothesis that calcified cartilage is a tissue very prone to cellular and nuclear preservation^6^. It is likely that innumerable other examples of preserved chondrocyte nuclei in Mesozoic material are waiting to be discovered.

The H&E staining in *Caudipteryx* revealed cellular structures that (1) share similar morphological characteristics and (2) stain with the same pattern as those in avian chondrocytes (i.e., cytoplasm, nuclear membrane, and a nucleus with condensed chromatin). Proposing a contamination of this dinosaur cell by a chromatin mimicking structure or by a chromatin shaped organism is not scientifically sound. Based on the data provided by this study, on existing data on extant cartilage from forensics studies (e.g., ref. ^23^), existing data on nuclear fossilization in both the Phanerozoic^2^ and specifically in other dinosaur cartilage material^6^ and dinosaur bone material^35^, the most logical conclusion is that the H&E staining is binding to endogenous structures and that this *Caudipteryx* cell preserves its cytoplasm and its nucleus. The chromatin threads do not show any clearly identifiable mitotic conformation (i.e., from neither prophasic, metaphasic, anaphasic, nor telophasic chromosomes), therefore it is not possible to identify the specific cycle of this cell and it is safer to refer to these structures as fossilized threads of chromatin, not to chromosomes.

Based on qualitative histology seen in the ground-sections and the chemistry of the tissues and surrounding sediments shown by the EDS analyses (Fig. 2; Supplementary Notes 1, 2), it can be concluded that STM4-3 first underwent an event of alumino-silicification (most likely shortly after death and during early diagenesis), followed by a secondary invasion and alteration by iron that percolated through the cartilage and invaded the cracks and porous spaces of the subchondral bone. Many areas of the tissues of STM 4-3 (the subchondral bone, calcified cartilage, and the muscle tissue) are alumino-silicified (Supplementary Notes 1–3), suggesting that the main factor that allowed nuclear preservation in this specimen is a permineralization by silicification. Because this specimen is still relatively well-articulated and has many traces of soft tissues, permineralization may have occurred within a few days to a few weeks after death, before chondrocyte autolysis could start. STM4-3 represents the fifth example of Jehol fossil with alumino-silicified tissues. Indeed, alumino-silicification was also observed in the remnants of the perifollicular membrane of ovarian follicles in an enantiornithine (STM 10-12)^20^, in some clam shrimp eggs^36^, in the soft-tissues surrounding the dentary of *Yanornis* (IVPP V13358)^18^ and in the furcular chondrocytes of *Confuciusornis* (IVPP V11521)^18^. This confirms that alumino-silicification is a common mechanism of skeletal and soft tissue preservation in the Jehol biota (*contra*^37^). Alumino-silicates are found in the sediments near the fragment that was extracted (Supplementary Notes 1, 2) and are common in the Jehol (e.g., ref. ^16^).

The ironization of this specimen seems to have been a later diagenetic event where iron percolated only through parts of the cartilaginous and bony ECMs, precipitated around some of the cells (Fig. 3d; Supplementary Fig. 2) and infiltrated through breaks between bony trabeculae. Iron did not infiltrate the alumino-silicifed muscle tissue (Supplementary Note 3), and this may be because the muscle fibers were too tightly packed and without breaks for any infiltration to be possible. Alternatively, it is possible that iron has a chemical affinity with hydroxyapatite (present in bone and cartilage, but absent in muscle). The source of this iron is unknown, but it is highly likely that it comes from ground waters and precipitated into the tissues.

It has been hypothesized that iron oxides are involved in the preservation of cells in Mesozoic material because they often form a sheath around fossil bone cells^38,39^. Here, an iron sheath was also revealed around the cartilage cells of *Caudipteryx* in unstained paraffin sections (Fig. 3d); and a similar sheath of iron oxides was also observed around a few bone cells in TEM ultrathin sections (Supplementary Fig. 2). Iron sheaths surrounding fossilized cartilage cells were never reported before and were not observed in the well-preserved chondrocytes of *Hypacrosaurus*^6^. However, since the chromatin threads that we report here in *Caudipteryx* were identified specifically in a non-ironized cell, this suggest iron may not always play a role in cellular nor nuclear preservation. It is possible that iron oxides have an affinity for elements of the cell membrane and precipitate around them, but this precipitation may not necessarily occur during early diagenesis, which is a key period for exceptional fossilization (e.g., ref. ^40^).

The ground-sections of the *Caudipteryx* femur fragment showed bone and cartilage that were invaded by a large amount of dark mineralization preventing any clear viewing of the tissues and cells under transmitted light (Fig. 2a, c) and polarized light (Fig. 2d–f). The exquisite cellular preservation seen in the cartilage of this specimen after demineralization and transformation of the sample into paraffin sections (Fig. 3) could not have been assessed only using ground-sections. This shows that to fully grasp the type of preservation in any fossil tissue, multiple complementary methods should always be used together as proposed before (e.g., ref. ^20^). Paraffin histology applied to demineralized fossil tissues and paired with standard histochemical stains has proven useful to identify two types of bone tissues in *Tyrannosaurus rex*^41^, bone and cartilage in the skulls of the ornithuromorph *Yanornis*^15^ and the dinosaur *Hypacrosaurus*^6^, fossilized muscle fibers and collagen fibers in an enantiornithine^20^, and here a cartilage cell nucleus in *Caudipteryx*. A similar method using another stain (the Feulgen stain) which binds to the sugar-phosphate backbone of the DNA molecule was successfully applied to Eocene leaf and fruit tissues and clearly stained the nuclei in red^42,43^. More recently, some exquisitely preserved plant anaphasic chromosomes from the Oligocene were stained using this same histochemical method and the staining was clearly located within the chromosomes only (not within the cytoplasm nor the cell membrane), demonstrating that the staining was specific and that the method worked^7^. Other examples of histochemical stains were applied to a variety of fossils tissues and cells from different localities, again showing a similar reactivity in the fossil tissues when compared to analogous extant tissues (e.g., ref. ^44^). In the present study, histochemistry has helped discover structures that were unobservable in the thicker ground-sections. All of this published, abundant evidence shows that recent claims that histochemistry is inappropriate for fossil tissues^37^ are completely invalid and unsupported. One important factor that must be noted is that sediments and other non-original, non-biological structures can be stained and show non-specific binding, therefore it is important to correctly identify specific from non-specific binding (Supplementary Note 8).

In extant tissues, the precise interaction and staining mechanism of Hematoxylin with the nucleic acids of cell nuclei and Eosin with intra- and extracellular proteins is still incompletely understood^19^. Even though hematoxylin binds to DNA in extant cells, H&E alone cannot be considered a powerful enough stain to suggest that ancient DNA is preserved in this fossilized material (for this, more specific DNA stains like the Feulgen stain, or DNA fluorescent dyes like Propidium Iodide or DAPI would be preferred; e.g., refs. ^6,7,35^). The H&E staining observed here does however show that differences in chemistries between the chondrocyte cytoplasm and the nucleus were preserved for millions of years, still retaining the ability to properly interact with standard histochemical stains. It also shows that some of the original nuclear biochemistry is preserved in this dinosaur cartilage material. It was recently proposed that even though DNA is apparently in a non-PCR amplifiable and non-sequenceable form in Mesozoic fossils, some of the original chemistry and molecules may still be preserved in the form of DNA fossilization products^34^. This may explain why some dinosaur cells can still react with DNA stains^6,35^ even though a DNA sequence has never been authenticated in any fossil much older than ~1.2 My^45^. Although the results presented here are preliminary chemical data, they still support the hypothesis concerning DNA fossilization products^34^ and reaffirm that much more efforts need to be made to investigate all the unanswered questions about DNA preservation in deep time, especially in fossilized cartilage.

## Methods

### Sample preparation

A piece of distal femur (articular surface) was extracted from STM4-3 using a razor blade and a hammer. This original sample was then further broken into three smaller pieces with the same tools (Fig. 1b). One fragment was embedded in resin and transformed into ground-sections and observed under the light microscope and the SEM/EDS. The second fragment was embedded in agar, decalcified for 21 days and processed into both natural (unstained) and stained paraffin slides. The third fragment was embedded in SPI-PON 812 Resin, trimmed and cut into ultrathin sections (70 nm) using a diamond knife for the TEM and STEM imaging and element analyses (Supplementary Note 7).

### Petrographic ground-sections

One of the fragments was embedded in EXAKT Technovit 7200 (Norderstedt, Germany) one-component resin, and cured for 24 h, cut using an EXAKT 300CP accurate circular saw, and then ground and polished using the EXAKT 400CS grinding system (Norderstedt, Germany) until the desired optical contrast was reached (about 30 μm). Sections were observed under transmitted and polarized light using a Nikon eclipse LV100NPOL and photographed with a DS-Fi3 camera and the software NIS-Element v4.60.

### SEM-EDS

The ground-sections were analyzed using the SEM at the Chinese Academy of Geological Sciences (CAGS) with a FEI Quanta 450 (FEG) at 20 kV. Both BSE and SE modes (backscattered electrons and secondary electrons) were applied. The EDS profiles were measured as maps (Fig. 2 and Supplementary Notes 1–3).

### Decalcification and unstained paraffin sections of *Caudipteryx*

One of the fossil cartilage fragments was embedded in 3% agar (Becton Dickinson Cat# 214530) (for stabilization of tissues) and decalcified in 500 mM EDTA; pH 8.0 for 21 days with a solution change every ~2–3 days. During demineralization, the agar-embedded fragment was cut into smaller pieces with sterile razor blades and re-embedded in agar to increase sample number (i.e., the number of paraffin blocks). After decalcification, the fragments were subjected to routine dehydration, clearing in xylene, and paraffin infiltration and embedding (Supplementary Note 7). Sections were cut at either 5 or 10 μm on a rotary microtome (Leica Biosystems RM2265), placed into a warm water bath (at about 44 °C) and mounted on charged slides (Superfrost Plus, Fisher Scientific). Most slides of STM4-3 were left unstained: they were simply deparaffinized in different solutions of xylene for about 15 min and cover-slipped with mounting medium (Permount, Fisher Scientific). Sections were observed under transmitted light using a Nikon eclipse LV100NPOL and photographed with a DS-Fi3 camera and the software NIS-Element v4.60.

### SEM-EDS on decalcified cells of *Caudipteryx*

One *Caudipteryx* paraffin slide (Fig. 3f) was deparaffinized in xylene, then left to air dry for a few minutes and put into a protective box (without any coverslip). It was then sprayed with gold at CAGS and observed with an FEI Quanta 450 (FEG) at 20 kV. Both BSE and SE modes were applied. The EDS profiles were measured as points (Fig. 3g–i; Supplementary Notes 4–6).

### Decalcification and paraffin sections of *Gallus*

A piece of proximal articular cartilage was dissected and extracted from a frozen adult chicken obtained legally and commercially (a gravid hen). The fragment was then fixed in 10% Neutral Buffered Formalin for 48 h, then demineralized in a solution of 20% HCl and 30% formic acid (JYBL-II, Cat DD0017, Leagene) for ~48 h. After this, the demineralized fragment was processed for paraffin histology with the exact same protocol as that listed in the previous section for *Caudipteryx* (Supplementary Note 7 for details).

### H&E staining

A few slides of STM4-3 (5 μm thick) and of the extant chicken (also 5 μm thick) were stained with the standard nuclear stain H&E from a pre-made commercial kit (Beijing SolarBio Life Sciences, kit G1120) (see Supplementary Note 7 for full protocol). Two different sets of staining dishes and SolarBio kits were used for the fossil and the extant slides to avoid contamination (Supplementary Note 7). Stained sections were observed under transmitted and polarized light using a Nikon eclipse LV100NPOL and photographed with a DS-Fi3 camera and the software NIS-Element v4.60 (with immersion oil for Fig. 4g, h). Only two images were photographed with another microscope (a Nikon Eclipse Ni with a DS-Ri2 camera) at ×60 (Fig. 4c, e).

### Reporting summary

Further information on research design is available in the Nature Research Reporting Summary linked to this article.

## Supplementary information

Supplementary Information

Reporting summary

## Data Availability

STM4-3 is reposited at the Shandong Tianyu Museum of Nature in Linyi City. All section types of the femur fragment of STM4-3 (ground, paraffin, and TEM sections) are currently reposited at the Institute of Vertebrate Paleontology and Paleoanthropology in Beijing. All data are available upon reasonable request.

## References

[1] Pang K (2013). The nature and origin of nucleus‐like intracellular inclusions in Paleoproterozoic eukaryote microfossils. Geobiology.

[2] Bailleul AM (2021). Fossilized cell nuclei are not that rare: review of the histological evidence in the Phanerozoic. Earth Sci. Rev..

[3] Darrah WC (1938). A remarkable fossil Selaginella with preserved female gametophytes. Botanical Mus. Leafl., Harv. Univ..

[4] Bomfleur B, McLoughlin S, Vajda V (2014). Fossilized nuclei and chromosomes reveal 180 million years of genomic stasis in royal ferns. Science.

[5] Yin Z (2017). Nuclei and nucleoli in embryo-like fossils from the Ediacaran Weng’an Biota. Precambrian Res..

[6] Bailleul AM (2020). Evidence of proteins, chromosomes and chemical markers of DNA in exceptionally preserved dinosaur cartilage. Natl Sci. Rev..

[7] Ozerov IA, Zhinkina NA, Torshilova AA, Machs EM, Rodionov AV (2021). Chromosomes of fossilized Metasequoia from early Oligocene of Siberia. Rev. Palaeobot. Palynol..

[8] Carlisle EM, Jobbins M, Pankhania V, Cunningham JA, Donoghue PC (2021). Experimental taphonomy of organelles and the fossil record of early eukaryote evolution. Sci. Adv..

[9] Sun W (2020). Nucleus preservation in early Ediacaran Weng’an embryo-like fossils, experimental taphonomy of nuclei and implications for reading the eukaryote fossil record. Interface Focus.

[10] Brack-Hanes SD, Vaughn JC (1978). Evidence of Paleozoic chromosomes from lycopod microgametophytes. Science.

[11] Vishnu, M. In *Chromosomes today* (eds Darlington, C. & Lewis, K.) (1967).

[12] Lee J., Orr-Weaver T. Chromatin Encyclopedia of Genetics, Academic Press, 340–343 (2001).

[13] Zhou Z (2014). The Jehol Biota, an early cretaceous terrestrial Lagerstätte: new discoveries and implications. Natl Sci. Rev..

[14] Zhou Z, Barrett PM, Hilton J (2003). An exceptionally preserved lower Cretaceous ecosystem. Nature.

[15] Bailleul AM, Li Z, O’Connor J, Zhou Z (2019). Origin of the avian predentary and evidence of a unique form of cranial kinesis in Cretaceous ornithuromorphs. Proc. Natl Acad. Sci. USA.

[16] Jiang B (2017). Cellular preservation of musculoskeletal specializations in the Cretaceous bird *Confuciusornis*. Nat. Commun..

[17] Wu Q., O’Connor J. K., Li Z.-H., & Bailleul A. M. Cartilage on the furculae of living birds and the extinct bird *Confuciusornis*: a preliminary analysis and implications for flight style inferences in Mesozoic birds. *Vertebrata Palasiatica*, **59**, 106–124 (2021).

[18] Bailleul A. M. & Zhou Z. SEM analyses of fossilized Chondrocytes in the extinct birds *Yanornis* and *Confuciusornis*: insights on taphonomy and modes of preservation in the Jehol Biota. *Front. Earth Sci.*10.3389/feart.2021.718588 (2021).

[19] Fischer A. H., Jacobson K. A., Rose J., & Zeller R. Hematoxylin and eosin staining of tissue and cell sections. *Cold Spring Harb. Protoc.* **2008**, pdb. prot4986 (2008). 10.1101/pdb.prot4986 21356829

[20] Bailleul AM (2020). Confirmation of ovarian follicles in an enantiornithine (Aves) from the Jehol biota using soft tissue analyses. Commun. Biol..

[21] Feldman, A. T. & Wolfe, D. Tissue processing and hematoxylin and eosin staining. In *Histopathology*. Humana Press. 31–43 (Springer, New York, NY, 2014). 10.1007/978-1-4939-1050-2_3 25015141

[22] Fadloun A, Eid A, Torres-Padilla M-E (2013). Mechanisms and dynamics of heterochromatin formation during mammalian development: closed paths and open questions. Curr. Top. Dev. Biol..

[23] Bolton, S. N. Forensic taphonomy: investigating the post mortem biochemical properties of cartilage and fungal succession as potential forensic tools. http://hdl.handle.net/2436/579577 (2015).

[24] Paulis M, Hassan E, Abd-Elgaber N (2016). Estimation of postmortem interval from cartilage changes of rabbit auricle. Ain Shams J. Forensic Med. Clin. Toxicol..

[25] Rogers CJ (2011). Postmortem degradation of porcine articular cartilage. J. Forensic Leg. Med..

[26] Wiemann J, Crawford JM, Briggs DE (2020). Phylogenetic and physiological signals in metazoan fossil biomolecules. Sci. Adv..

[27] Oskam CL (2010). Fossil avian eggshell preserves ancient DNA. Proc. R. Soc. B: Biol. Sci..

[28] Mescher, A. *Junqueira’s Basic Histology: Text and Atlas*, Thirteenth Edition. (McGraw-Hill Education, 2013).

[29] Hall, B. K. *Bones and Cartilage: Developmental and Evolutionary Skeletal Biology* (Elsevier/Academic Press, 2005).

[30] Buckwalter J, Mankin H (1997). Articular cartilage: part I. J. Bone Jt. Surg..

[31] Buckwalter J, Mankin H (1997). Articular cartilage: part II. J. Bone Jt. Surg..

[32] Temenoff JS, Mikos AG (2000). Tissue engineering for regeneration of articular cartilage. Biomaterials.

[33] Lasczkowski GE, Aigner T, Gamerdinger U, Weiler G, Bratzke H (2002). Visualization of postmortem chondrocyte damage by vital staining and confocal laser scanning 3D microscopy. J. Forensic Sci..

[34] Bailleul AM, Li Z (2021). DNA staining in fossil cells beyond the quaternary: reassessment of the evidence and prospects for an improved understanding of DNA preservation in deep time. Earth-Sci. Rev..

[35] Schweitzer MH, Zheng W, Cleland TP, Bern M (2013). Molecular analyses of dinosaur osteocytes support the presence of endogenous molecules. Bone.

[36] Pan Y, Wang Y, Sha J, Liao H (2015). Exceptional preservation of clam shrimp (Branchiopoda, Eucrustacea) eggs from the Early Cretaceous Jehol Biota and implications for paleoecology and taphonomy. J. Paleontol..

[37] Mayr G, Kaye TG, Pittman M, Saitta ET, Pott C (2020). Reanalysis of putative ovarian follicles suggests that Early Cretaceous birds were feeding not breeding. Sci. Rep..

[38] Pawlicki R, Bolechała P (1991). X-ray microanalysis of fossil dinosaur bone: age differences in lead, iron, and magnesium content. Folia Histochem. Cytobiol..

[39] Schweitzer MH (2014). A role for iron and oxygen chemistry in preserving soft tissues, cells and molecules from deep time. Proc. R. Soc. B: Biol. Sci..

[40] Briggs DE (2003). The role of decay and mineralization in the preservation of soft-bodied fossils. Annu. Rev. Earth Planet. Sci..

[41] Schweitzer, M. H., Zheng, W., Zanno, L., Werning, S. & Sugiyama, T. Chemistry supports the identification of gender-specific reproductive tissue in *Tyrannosaurus rex*. *Sci. Rep.* **6**, 23099 (2016). 10.1038/srep23099 PMC4791554 26975806

[42] Ozerov IA, Zhinkina NA, Efimov AM, Machs EM, Rodionov AV (2006). Feulgen-positive staining of the cell nuclei in fossilized leaf and fruit tissues of the Lower Eocene Myrtaceae. Botanical J. Linn. Soc..

[43] Ozerov, I. A. et al. Use of DNA-specific stains as indicators of nuclei and extranuclear substances in leaf cells of the Middle Eocene *Metasequoia* from Arctic Canada. *Rev. Palaeobot. Palynol.* **279**, 104211 (2020).

[44] Koller B, Schmitt JM, Tischendorf G (2005). Cellular fine structures and histochemical reactions in the tissue of a cypress twig preserved in Baltic amber. Proc. R. Soc. B: Biol. Sci..

[45] van der Valk, T. et al. Million-year-old DNA sheds light on the genomic history of mammoths. *Nature* **591**, 265–269 (2021). 10.1038/s41586-021-03224-9 PMC7116897 33597750

