# Supplementary material for: Nuclear preservation in the cartilage of the Jehol dinosaur Caudipteryx
Source: Commun Biol. 2021 Sep 24;4:1125. doi: 10.1038/s42003-021-02627-8 (PMC8463611; doi:10.1038/s42003-021-02627-8)
Supplement: Supplementary file 1 — Supplementary Information [file 42003_2021_2627_MOESM1_ESM.pdf]

Supplementary Information for

Nuclear preservation in the cartilage of the Jehol dinosaur *Caudipteryx*

Xiaoting Zheng[1,2], Alida M. Bailleul[3,4*], Zhiheng Li[3,4], Xiaoli Wang[1,2], Zhonghe Zhou[3,4]

[1]Institute of Geology and Paleontology, Linyi University, Linyi City, Shandong 276005, China

[2]Shandong Tianyu Museum of Nature, Pingyi, Shandong 273300, China

[3]Key Laboratory of Vertebrate Evolution and Human Origins, Institute of Vertebrate Paleontology and Paleoanthropology, 142 Xizhimenwai dajie, Beijing, 100044, China.

[4]CAS Center for Excellence in Life and Paleoenvironment, Beijing 100044, China.

*Correspondence and requests for materials should be addressed to A.M.B (email: alida.bailleul@ivpp.ac.cn)

This PDF file includes:

Supplementary Figures 1 to 2

Supplementary Notes

Supplementary Note 1: EDS mapping of the tissues of STM4-3 and the adjacent sediments.

Supplementary Note 2: EDS mapping of the cartilage of STM4-3.

Supplementary Note 3: EDS mapping of a piece of muscle of STM4-3.

Supplementary Note 4: EDS report of a smooth silicified cell

Supplementary Note 5: EDS report of a porous silicified cell

Supplementary Note 6: EDS report of a striated, ironized cell

Supplementary Note 7: Supplementary Methods and Tissue Contamination Issues.

Supplementary Note 8: How to correctly interpret and use histochemical staining in fossil tissues and discussion on the issue of non-specific staining.

Supplementary References

Supplementary Figures

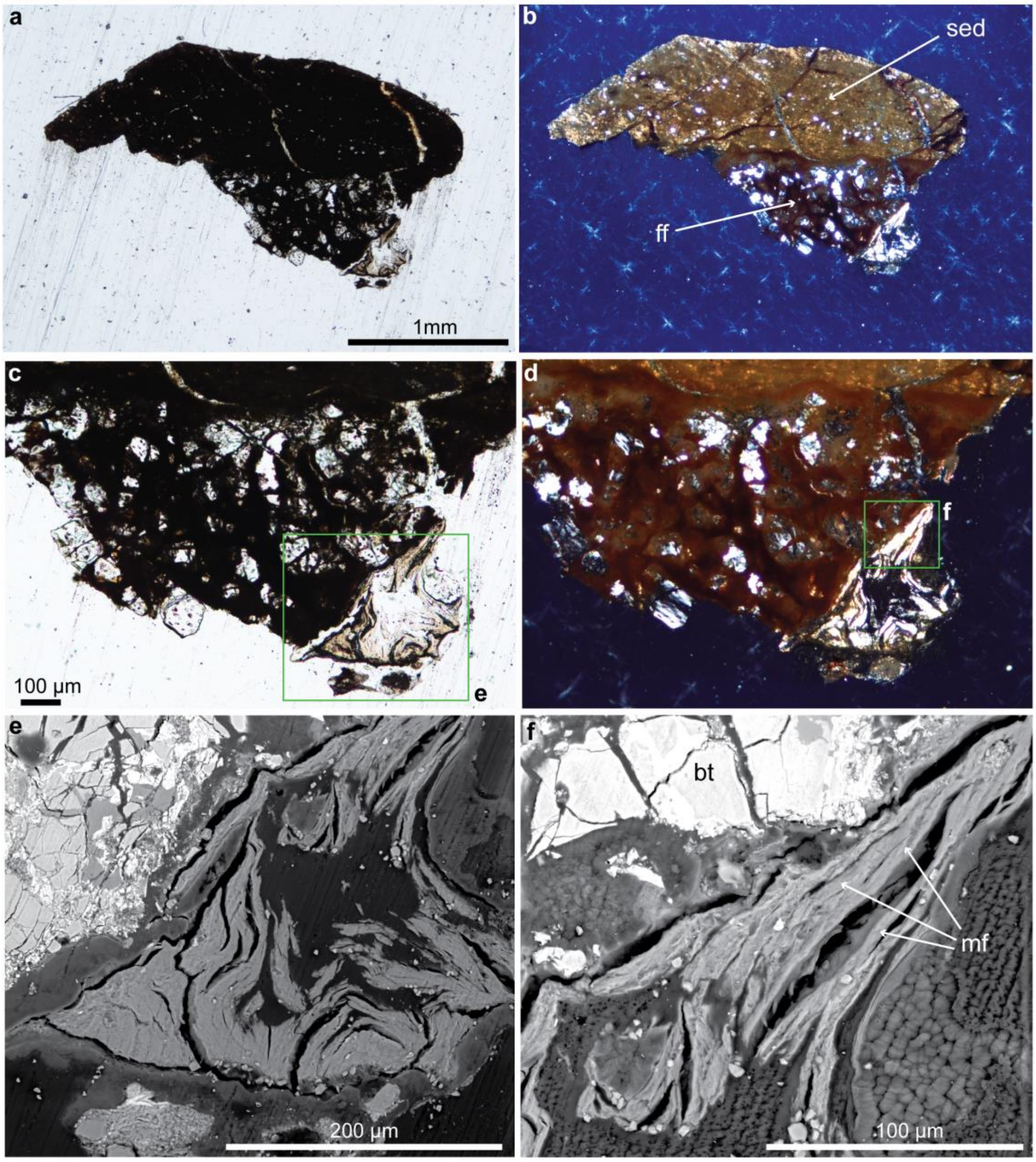

Supplementary Figure 1. Photographs of another ground-section of STM4-3 and SEM images. Photograph of ground-section under transmitted light (a). Same photograph but under the polarized light (b). Close-ups are shown in (c) and (d). A piece of muscle tissue can be seen as a light brown material under the transmitted light in (c) and is highly birefringent under the polarized light (d). SEM images of this piece of muscle are shown in (e) and (f), with clear muscle fibers (mf) identifiable. Abbreviations: bt, bone trabecula; ff, femur fragment; sed, sediment.

The birefringence of this fossilized muscle tissue is similar to that seen in the preserved perifollicular membrane (made mostly of muscle fibers as well) of the enantiornithine STM 10-12, also from the Jehol biota [1]. This is the second example of birefringent fossilized muscle tissue in the Jehol and suggests this is a very common characteristic of preserved soft-tissues when observed under the polarized light and in

ground-sections. Indeed, in extant tissues, birefringence occurs mostly in regularly arranged fibrillary tissues, such as collagen, muscle, or myelin[2] and in many other non-mineralized tissues. This goes against the argument of Mayr et al., [3] who suggested birefringence in the fossilized soft-tissues of STM10-12 was peculiar and more consistent with minerals. Birefringence is expected to be seen in muscle and collagenous tissues[2] whether or not they are fossilized.

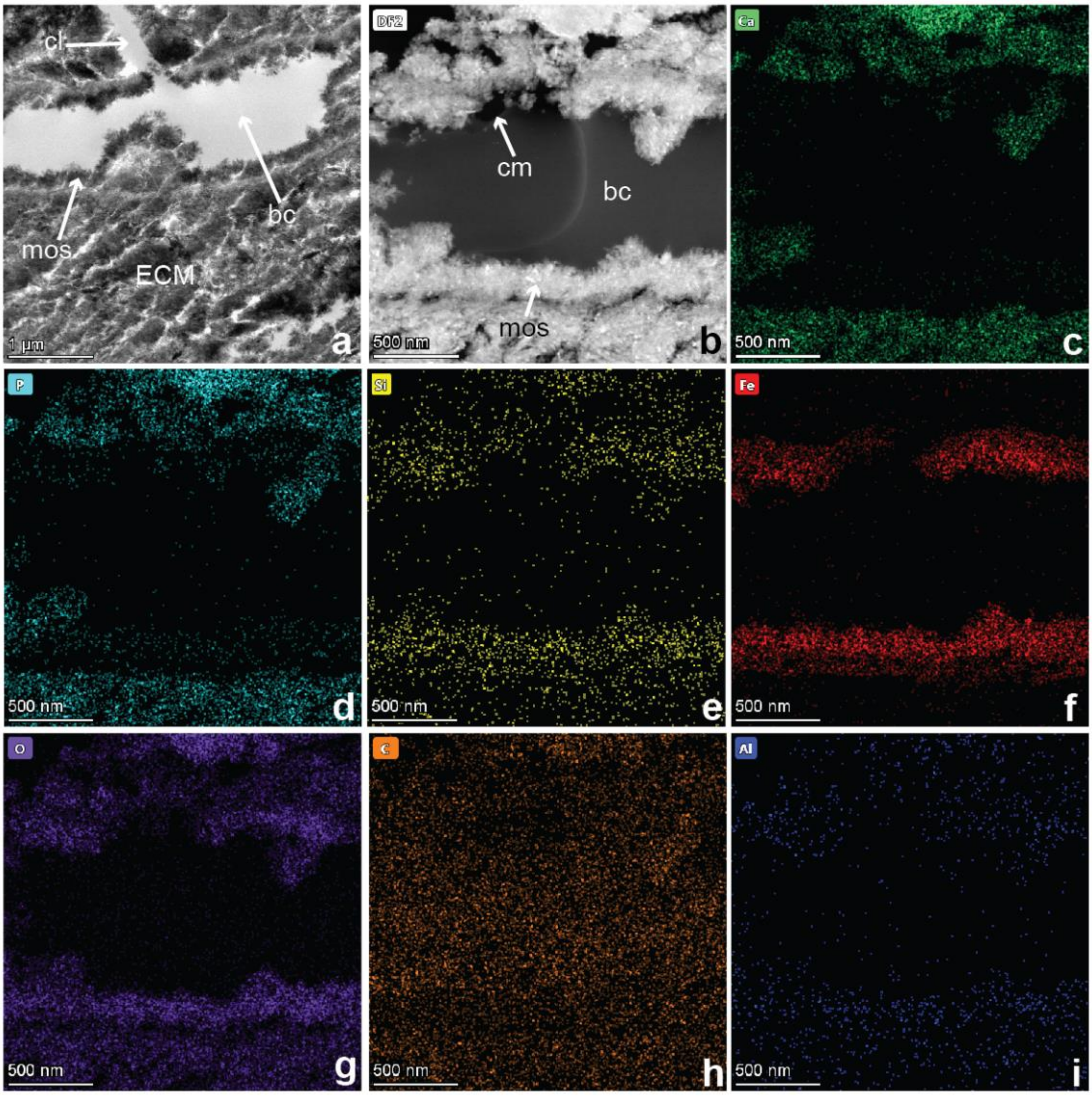

Supplementary Figure 2. Bright Field image (a), dark field image (b) with associated elemental mapping (c-i) of an ultrathin-section of STM4-3 taken using the Transmission Electron Microscope (FEI Talos 200X).

Bone extraceuular matrix (ECM) and bone cell lacuna filled with a bone cell (bc). A filopodia extending from the bone cell can be seen in a canaliculi (cl) (**a**). Another bone cell seen at higher magnification. The limit of the cell membrane (cm) can be seen (**b**). In both bone cells, no intracellular content can be seen, but they both have a metaloorganic sheath (mos) precipitated around their cell membrane. Elemental mapping of the main constituents of the cell seen in (b) are shown in (**c-i**). The bone cell is mostly made of carbon (therefore, it most likely is organic), and a few Silicon and Aluminum. The metaloorganic sheath is made of mostly of iron oxides and Silicon. We can hypothesize that this is the same type of sheath surrounding some of the cartilage cells in STM4-3 even though we could not be directly observed cartilage cells under the TEM.

Supplementary Note 1:

EDS mapping of the tissues of STM4-3 and the adjacent sediments.

Note that this is from the same ground-section as that shown in Supplementary Fig. 1 but flipped upside down (i.e., the sediments should be on top of the fragment, not below it as shown here). The image below shows a piece of muscle on the top left, then subchondral bone and calcified cartilage closer to the sediments.

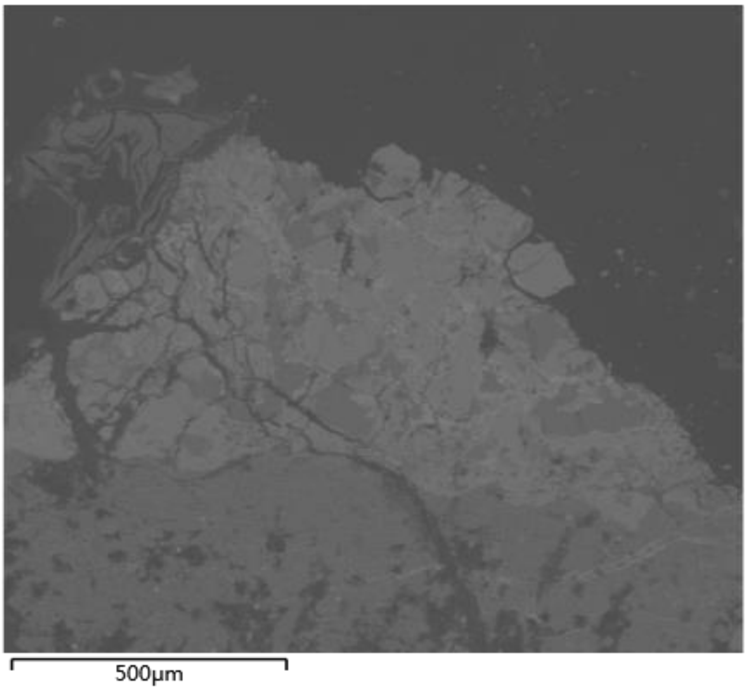

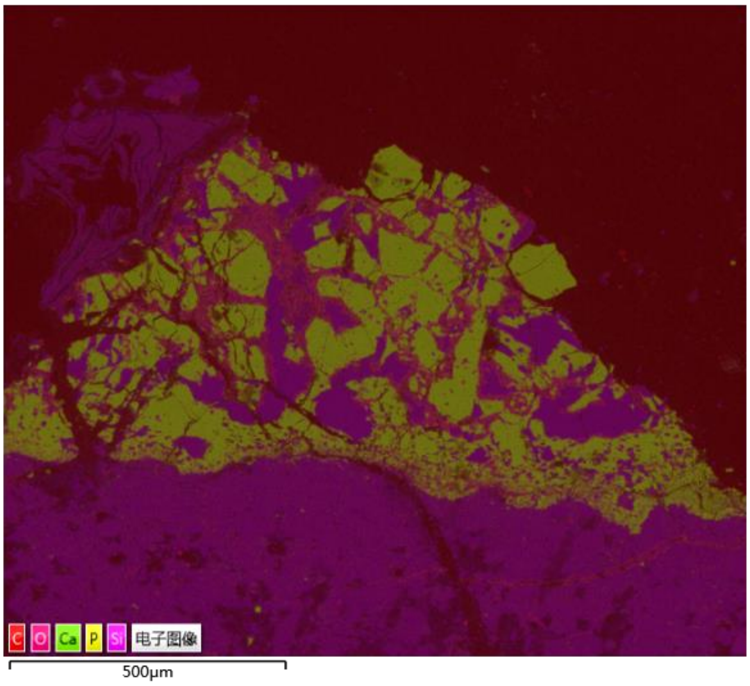

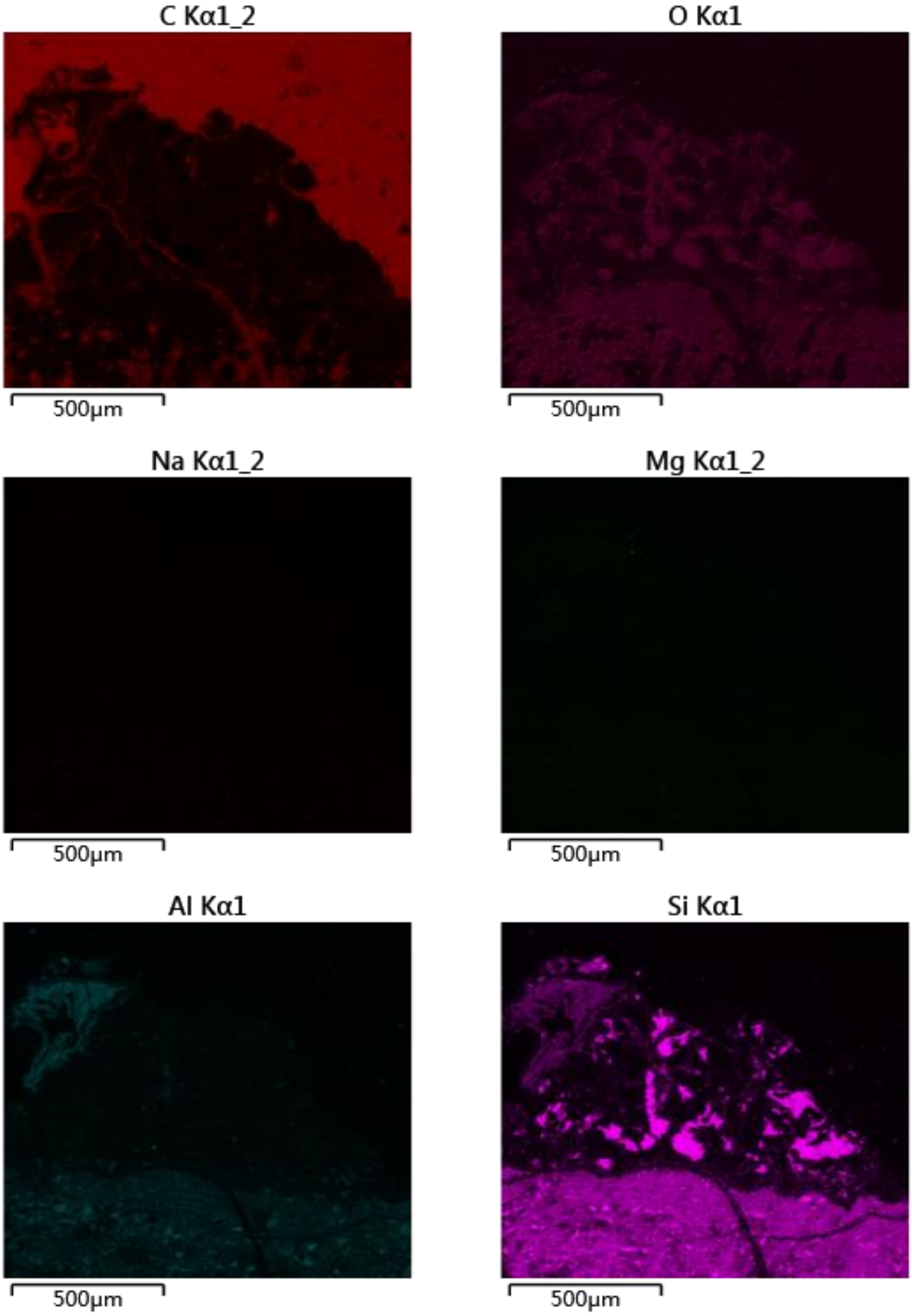
C Kα1_2
O Kα1
Na Kα1_2
Mg Kα1_2
Al Kα1
Si Kα1
500µm
500µm
500µm
500µm
500µm
500µm

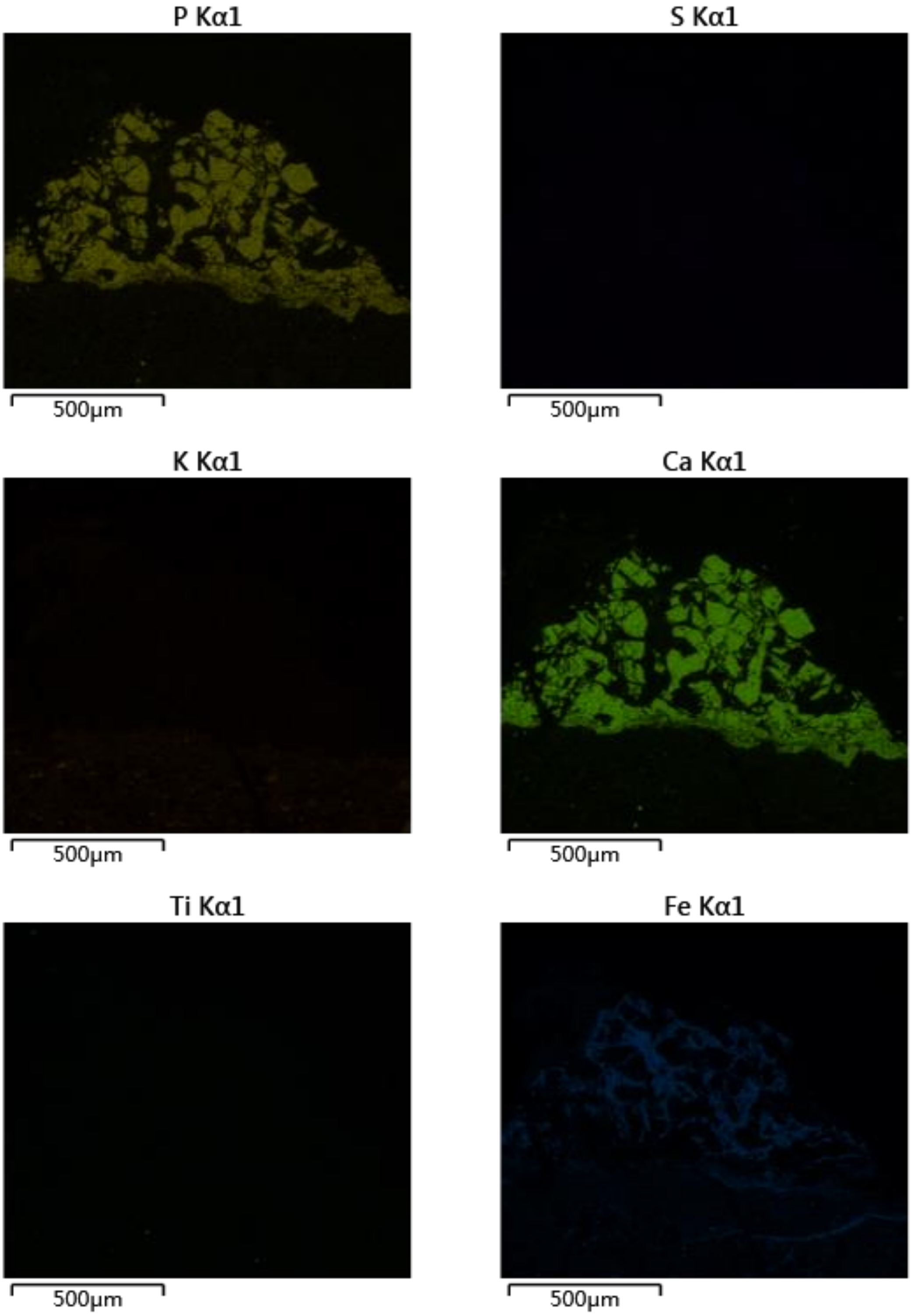
P Kα1
500µm
S Kα1
500µm
K Kα1
500µm
Ca Kα1
500µm
Ti Kα1
500µm
Fe Kα1
500µm

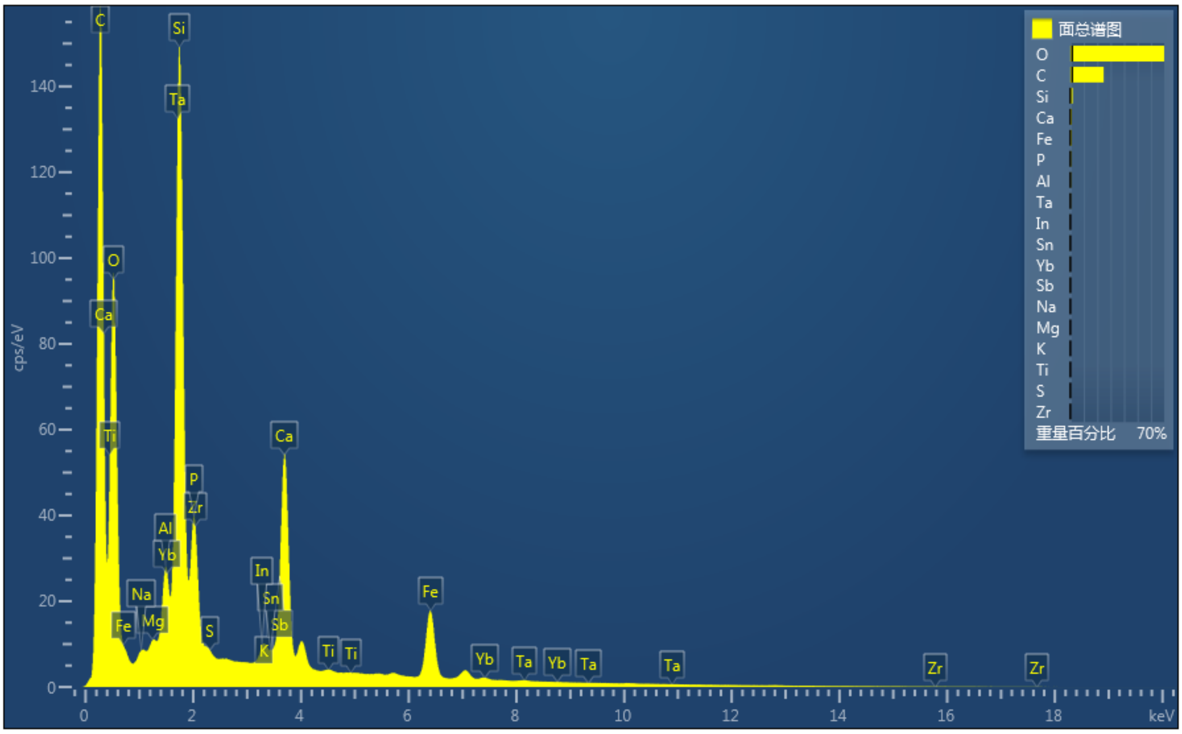

cps/eV
keV
面总谱图
O
C
Si
Ca
Fe
P
Al
Ta
In
Sn
Yb
Sb
Na
Mg
K
Ti
S
Zr
重量百分比 70%

Supplementary Note 2:

EDS mapping of the cartilage of STM4-3.

Note that this is the same image as that shown in Fig. 2g in the main manuscript, but flipped upside down (i.e., the sediments should be on top of the fragment, not below it as shown here). This Supplementary Note shows the full EDS mapping from this area to complete the selected elements (Si, Al and Fe) shown in the main manuscript.

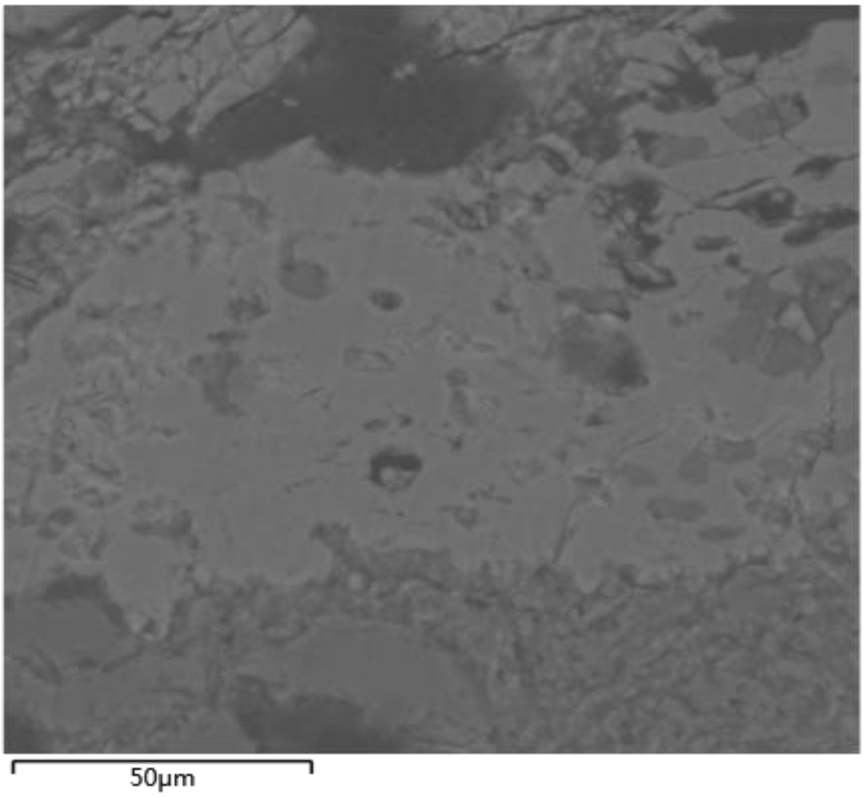

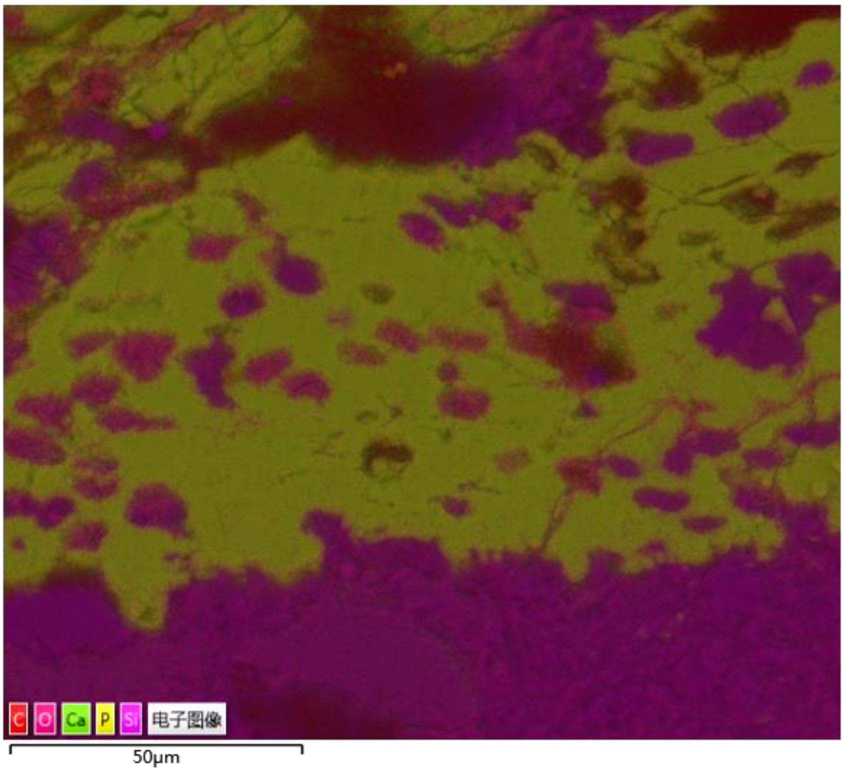
C
O
Ca
P
Si
电子图像
50μm

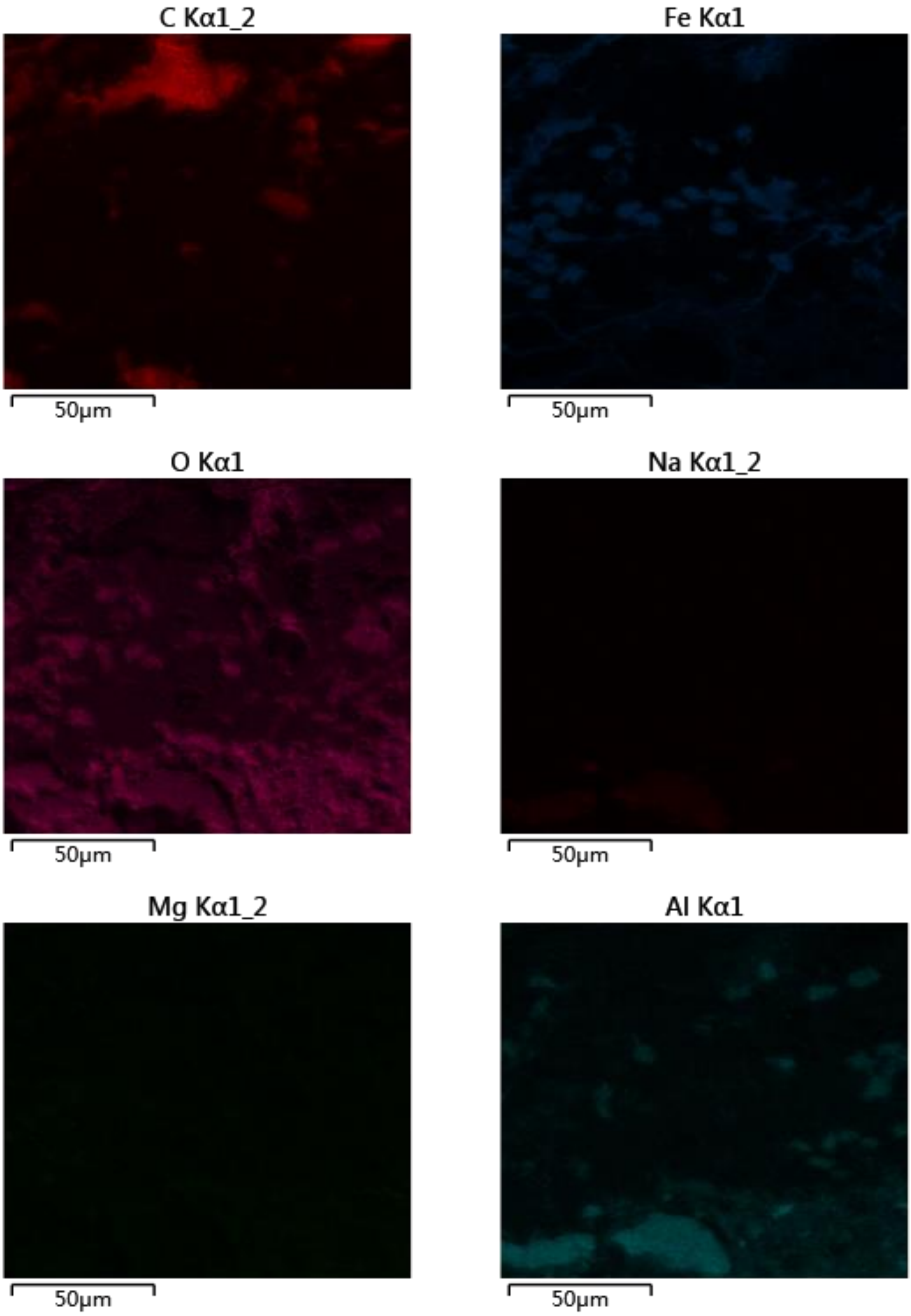
C Kα1_2
Fe Kα1
O Kα1
Na Kα1_2
Mg Kα1_2
Al Kα1
50µm

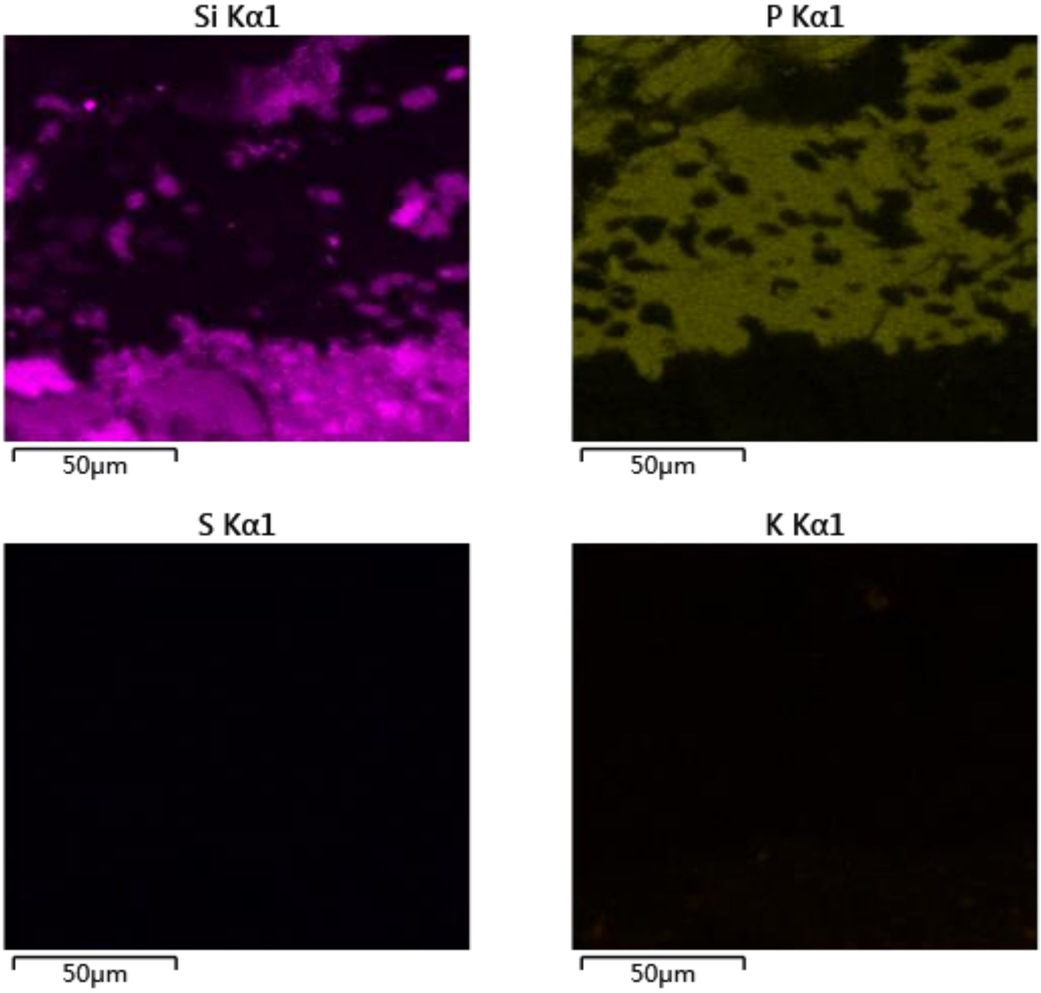
Si Kα1
P Kα1
50μm
50μm
S Kα1
K Kα1
50μm
50μm

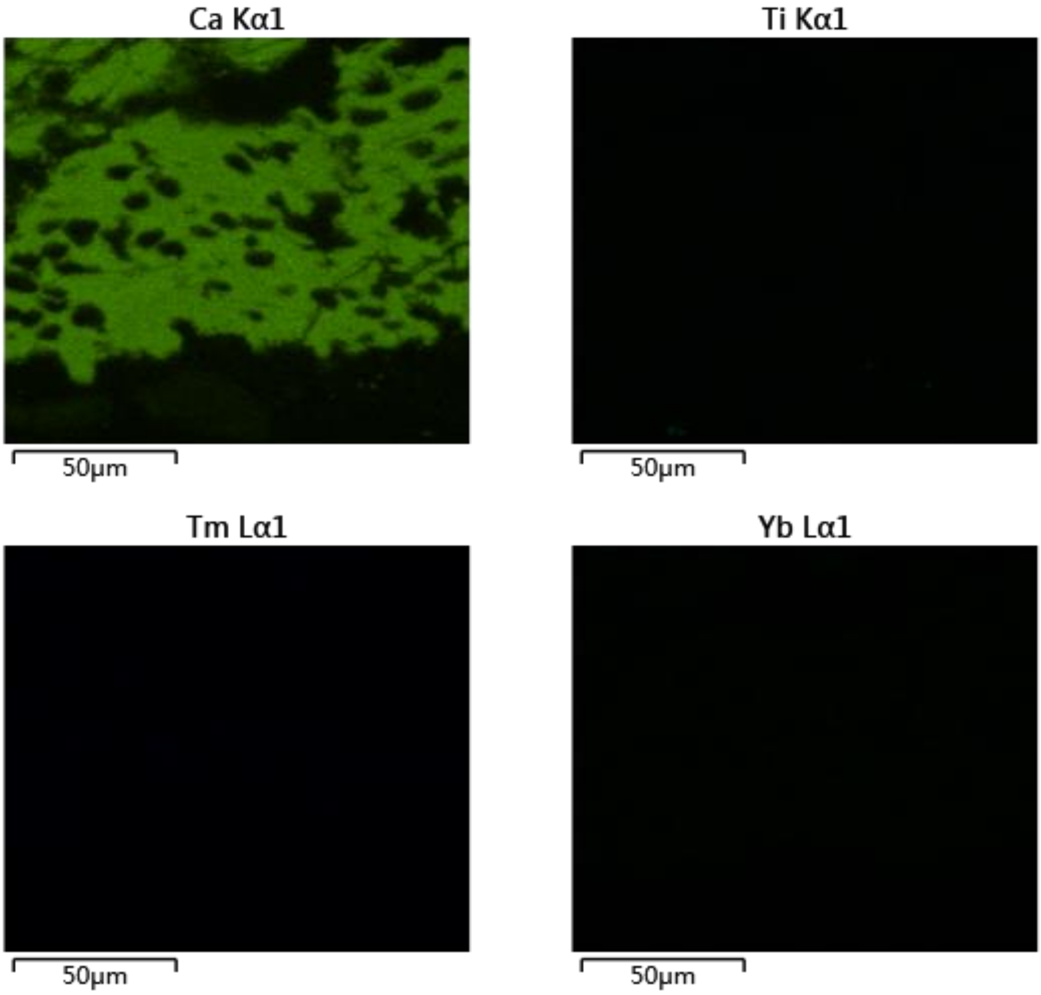
Ca Kα1
50μm
Ti Kα1
50μm
Tm Lα1
50μm
Yb Lα1
50μm

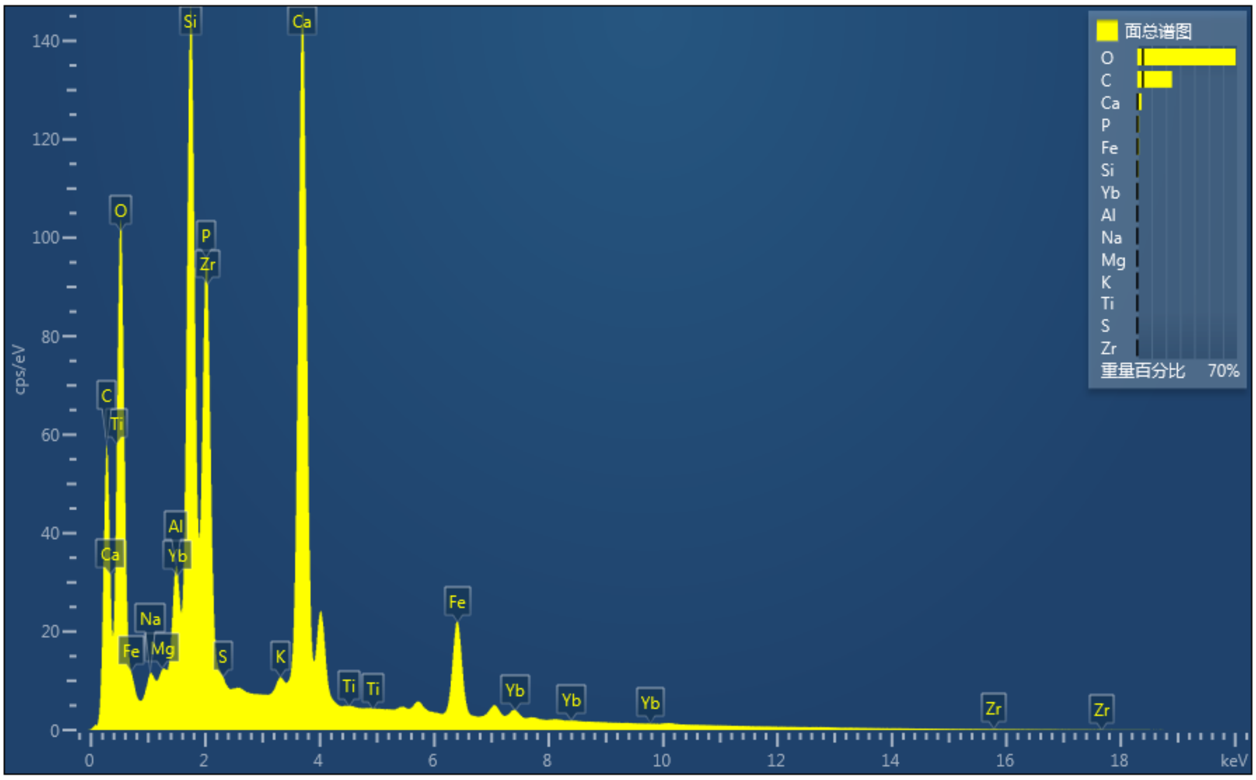

面总谱图
O
C
Ca
P
Fe
Si
Yb
Al
Na
Mg
K
Ti
S
Zr
重量百分比 70%
cps/eV
keV

Supplementary Note 3:

EDS mapping of a piece of muscle of STM4-3.

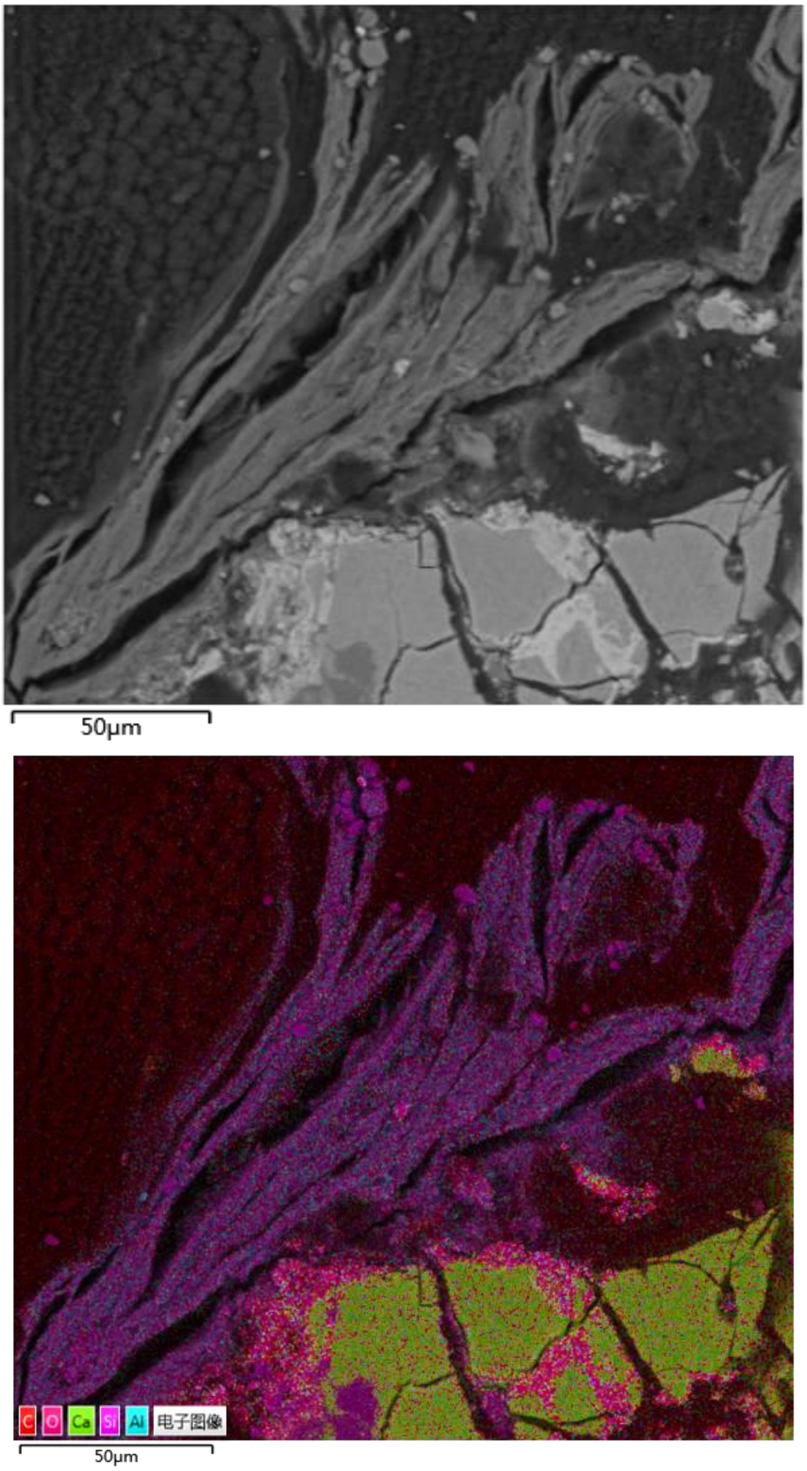

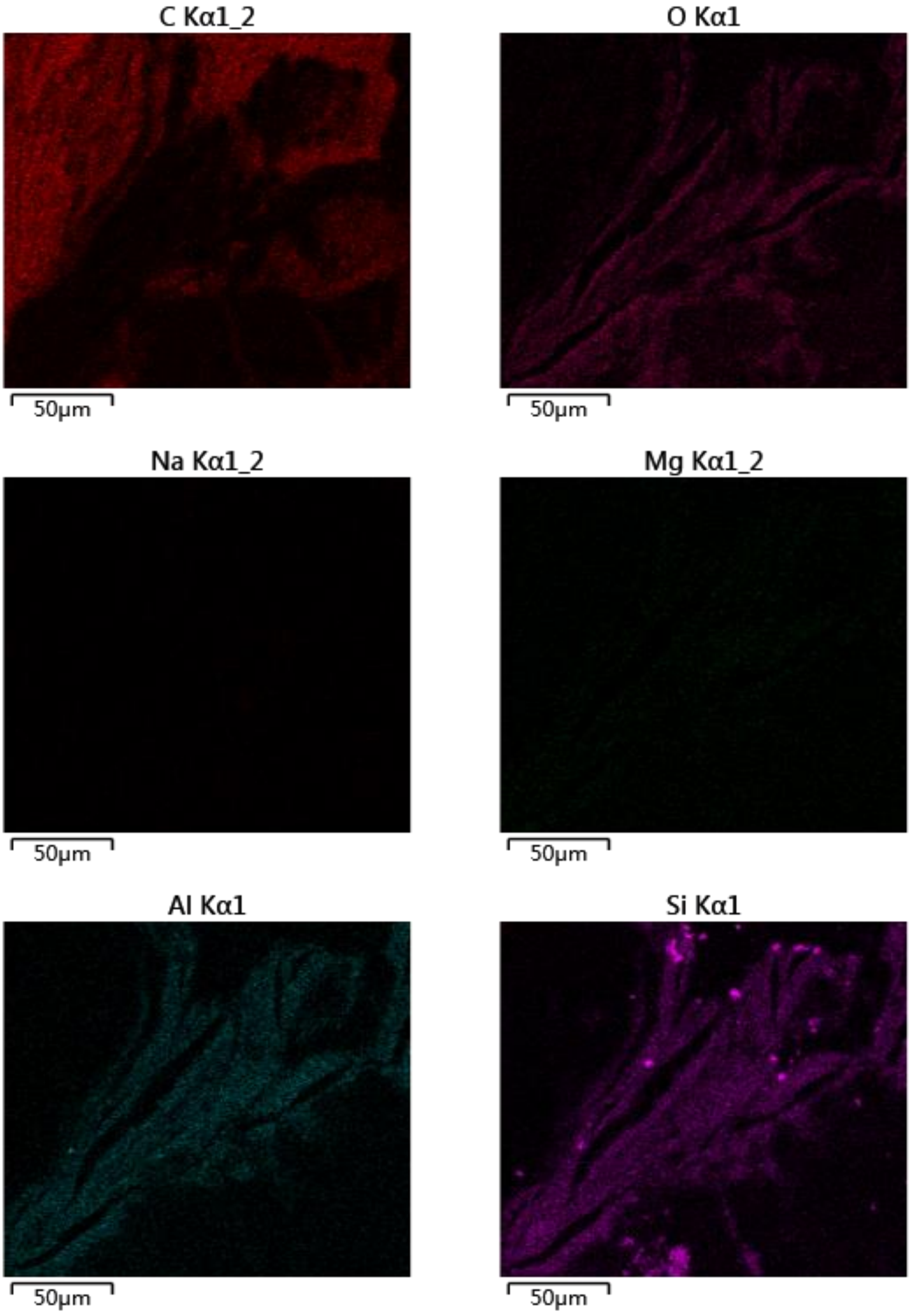
C Kα1_2
50µm
O Kα1
50µm
Na Kα1_2
50µm
Mg Kα1_2
50µm
Al Kα1
50µm
Si Kα1
50µm

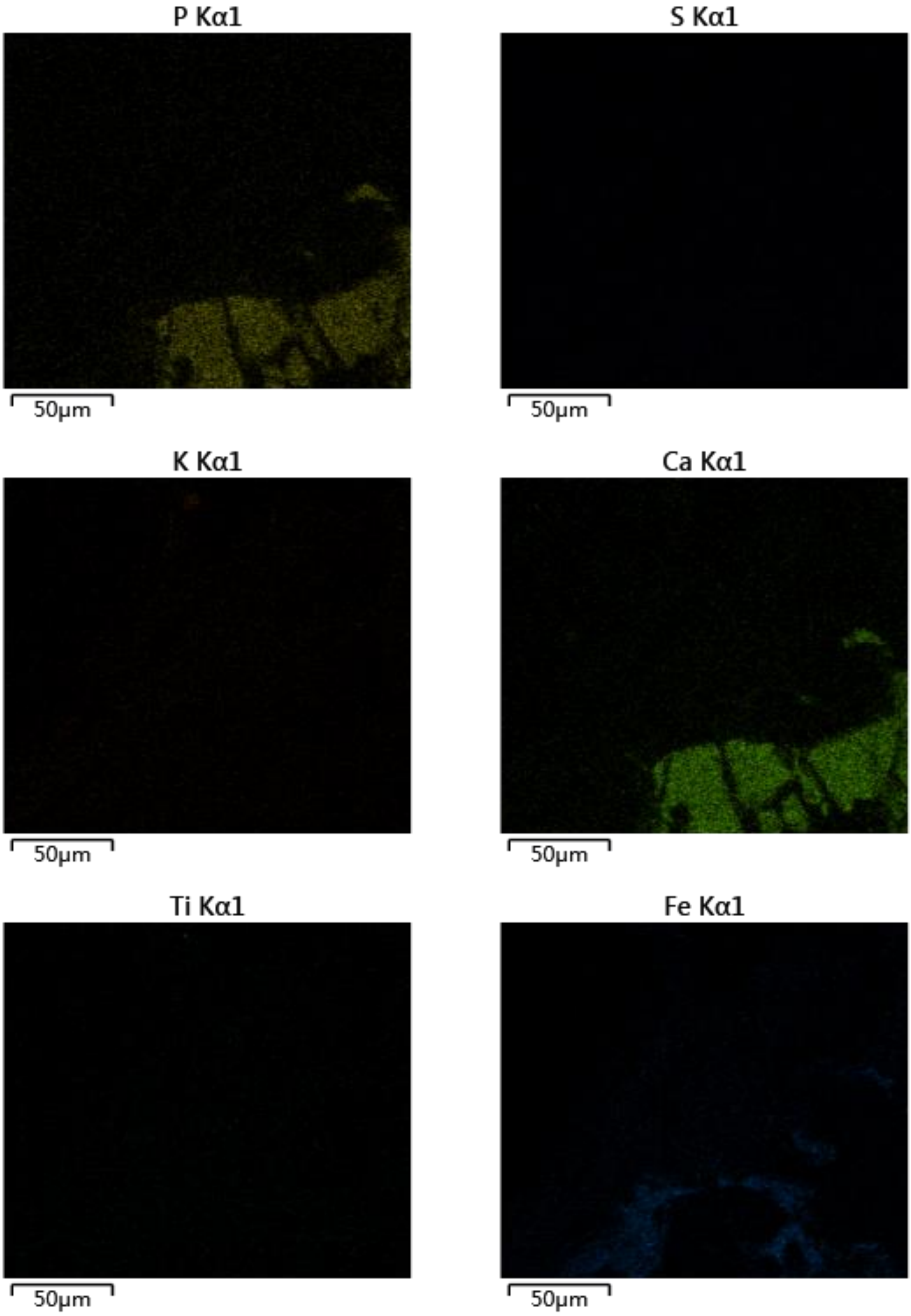
P Kα1
50μm
S Kα1
50μm
K Kα1
50μm
Ca Kα1
50μm
Ti Kα1
50μm
Fe Kα1
50μm

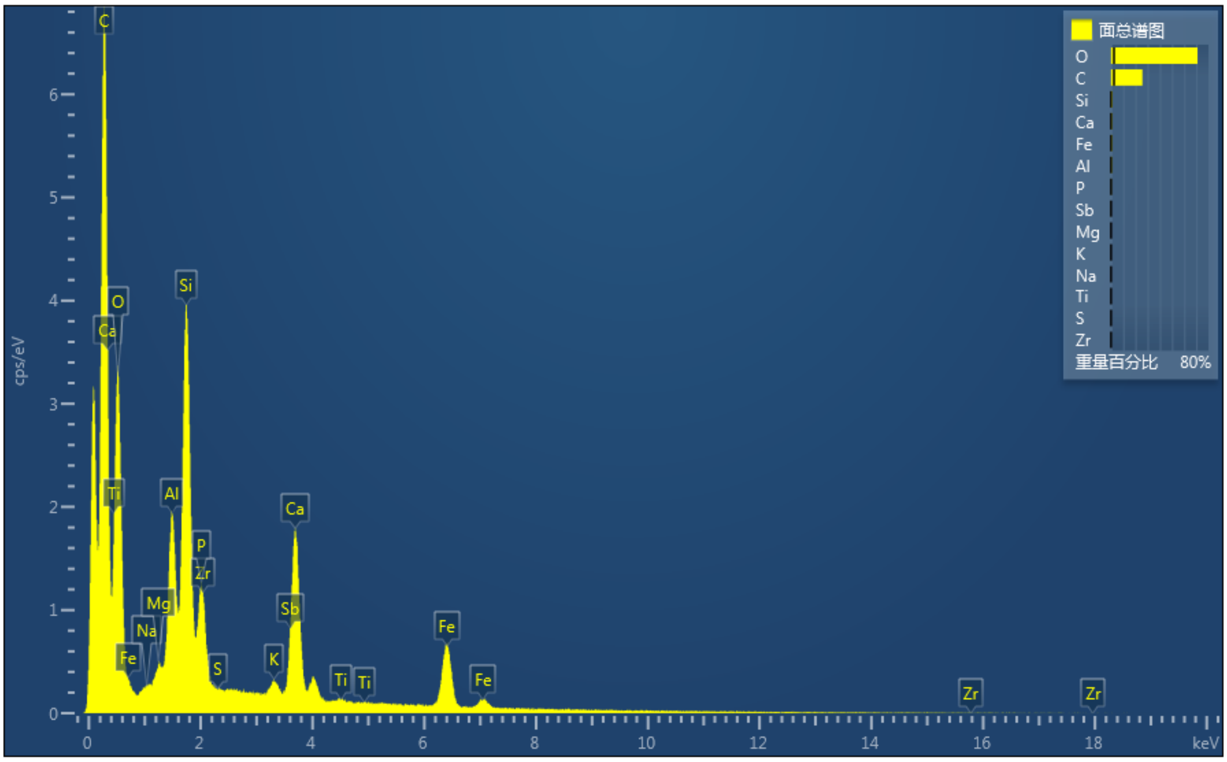

面总谱图
O
C
Si
Ca
Fe
Al
P
Sb
Mg
K
Na
Ti
S
Zr
重量百分比 80%
cps/eV
keV

Supplementary Note 4:

EDS report of a smooth silicified cell

Note that an EDS mapping could not be performed because the Silicon signal from the glass slide was blurring all the other elemental signals. Only EDS analyses by point were feasible.

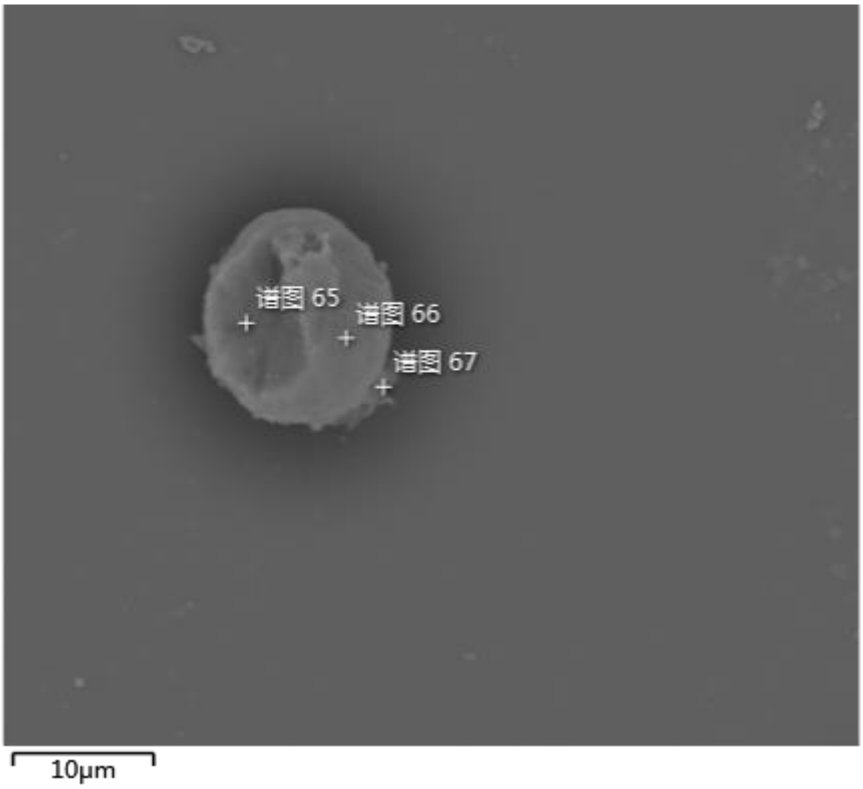

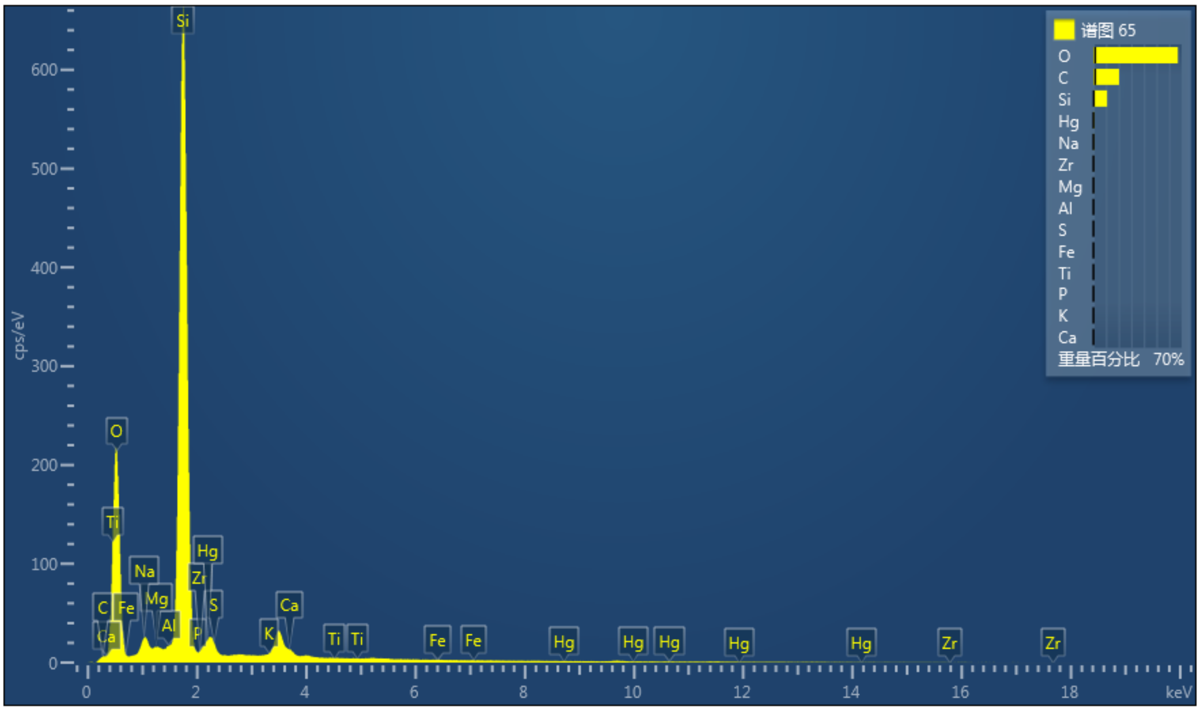

Elements	wt%	wt% Sigma
C	20.35	1.33
O	67.01	
Na	0.54	0.02
Mg	0.11	0.01
Al	0.06	0.01
Si	10.76	0.32
P	0.00	0.01
S	0.04	0.01
K	0.00	0.00
Ca	0.00	0.00
Ti	0.01	0.01
Fe	0.01	0.01
Zr	0.18	0.02
Hg	0.92	0.03
Total:	100.00	

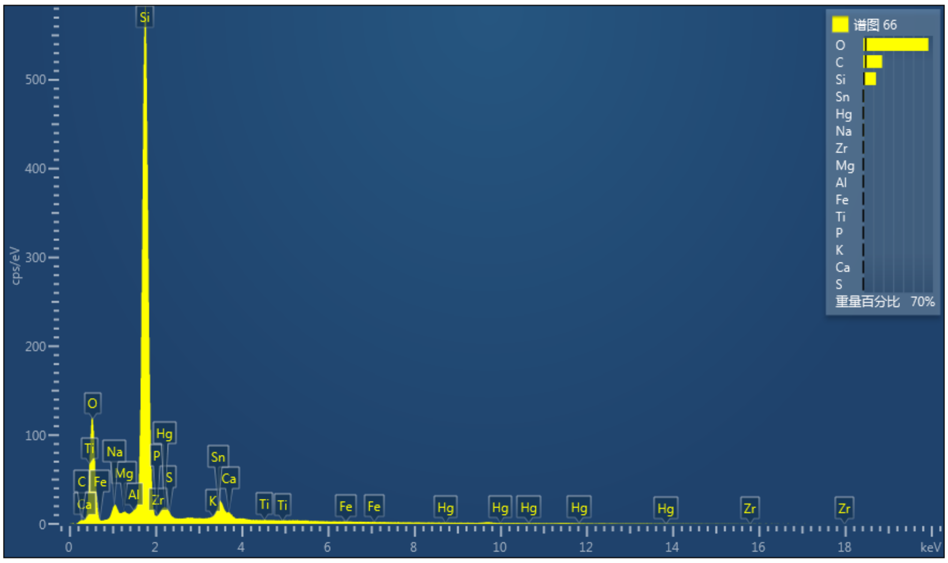

Elements	wt%	wt% Sigma
C	18.81	2.43
O	65.43	
Na	0.64	0.04
Mg	0.15	0.01
Al	0.10	0.01
Si	12.63	0.68
P	0.00	0.00
S	0.00	0.00
K	0.00	0.00
Ca	0.00	0.00
Ti	0.01	0.01
Fe	0.02	0.01
Zr	0.50	0.04
Sn	0.96	0.06
Hg	0.75	0.05
Total:	100.00	

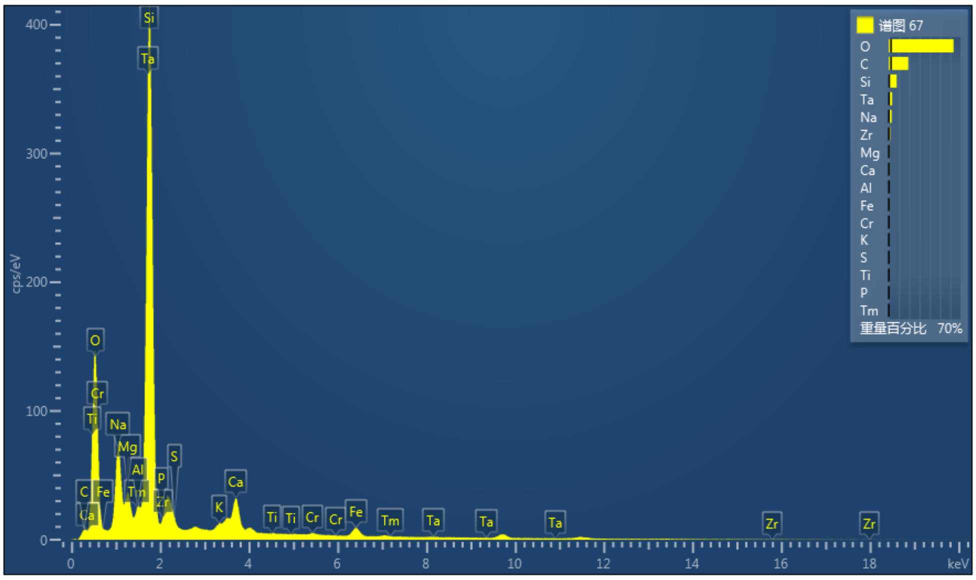

Elements	wt%	wt% Sigma
C	19.04	2.18
O	63.21	
Na	2.72	0.13
Mg	0.83	0.05
Al	0.58	0.09
Si	7.71	0.38
P	0.00	0.00
S	0.03	0.01
K	0.03	0.01
Ca	0.71	0.04
Ti	0.01	0.01
Cr	0.07	0.01
Fe	0.55	0.03
Zr	1.31	0.07
Tm	0.00	0.00
Ta	3.21	0.24
Total:	100.00	

Supplementary Note 5:

EDS report of a porous silicified cell

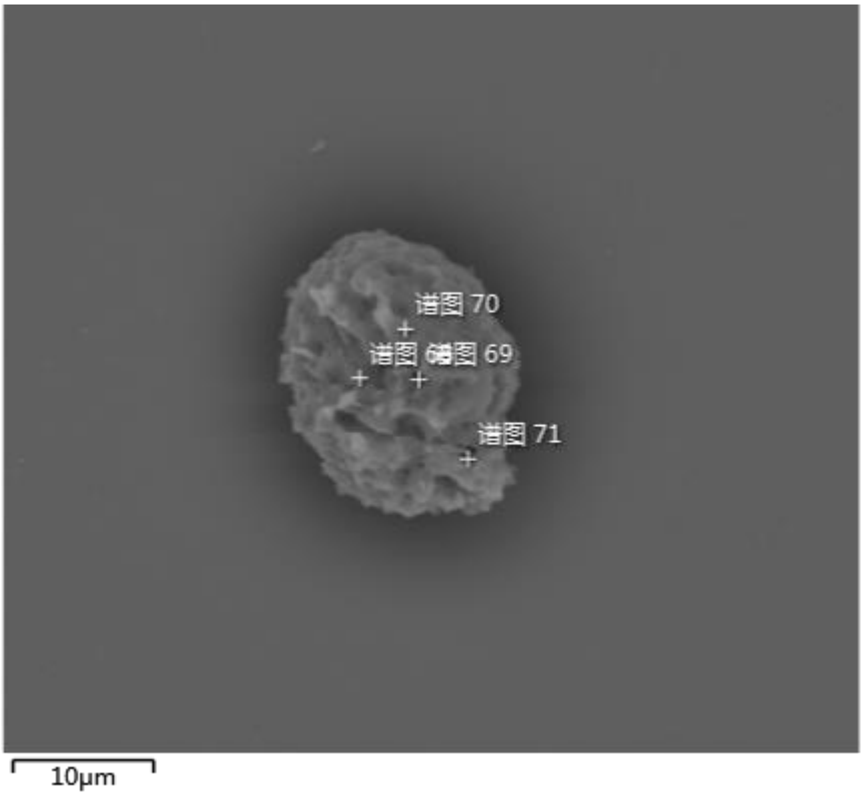

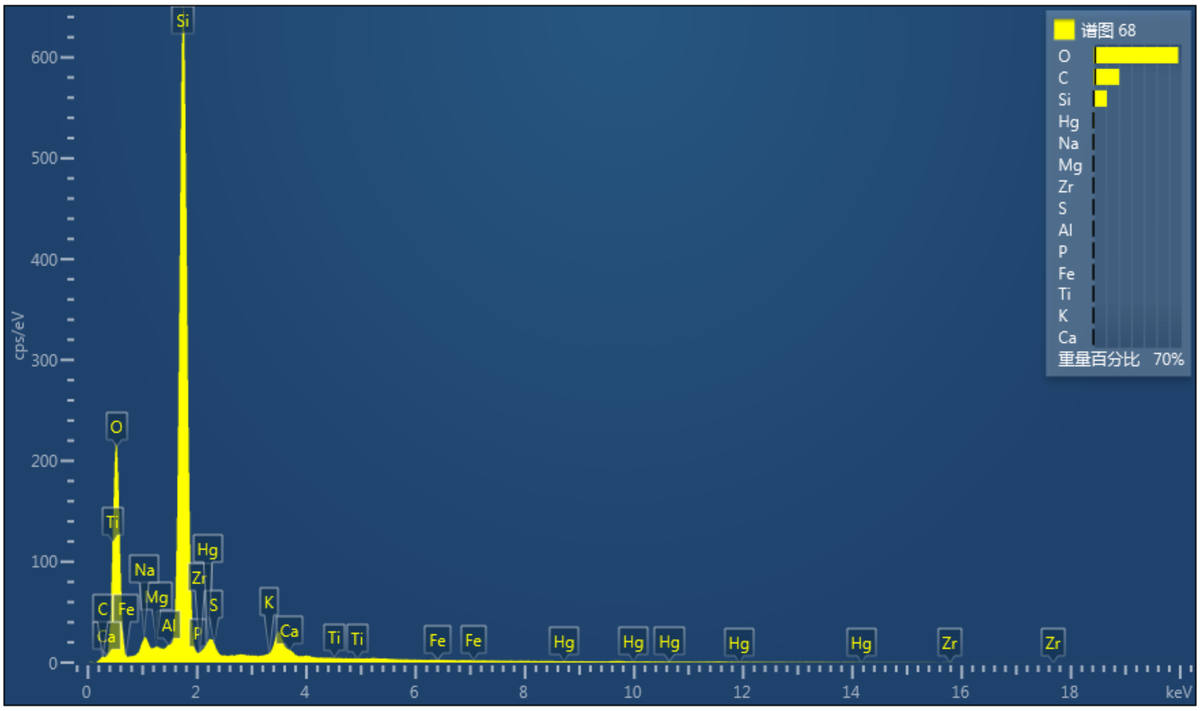

Elements	wt%	wt% Sigma
C	20.48	1.31
O	67.20	
Na	0.52	0.02
Mg	0.10	0.01
Al	0.06	0.01
Si	10.62	0.31
P	0.03	0.01
S	0.06	0.01
K	0.00	0.00
Ca	0.00	0.00
Ti	0.00	0.01
Fe	0.01	0.01
Zr	0.09	0.02
Hg	0.82	0.03
Total:	100.00	

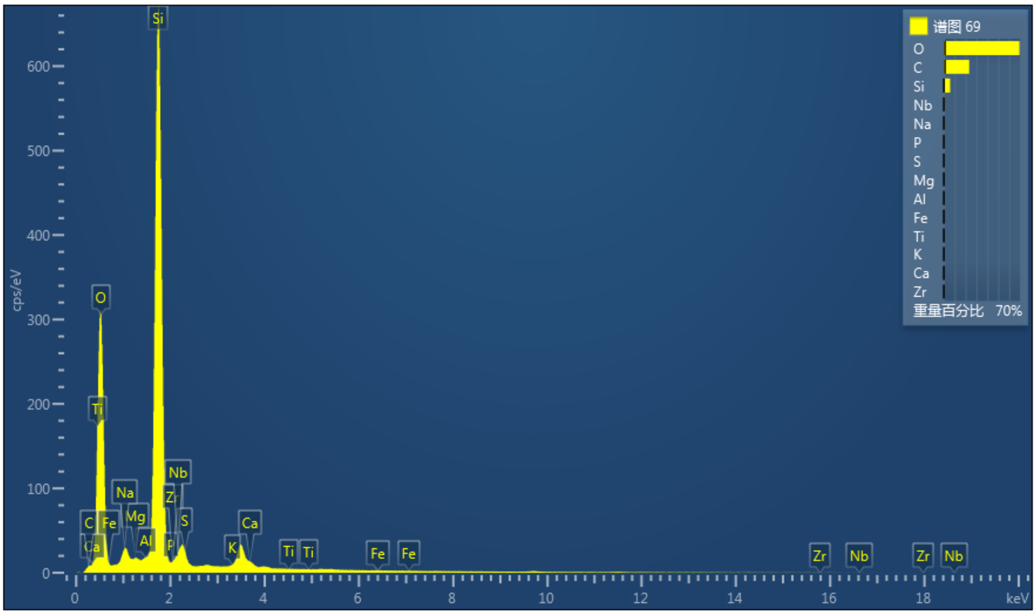

Elements	wt%	wt% Sigma
C	23.36	1.08
O	69.61	
Na	0.32	0.01
Mg	0.06	0.00
Al	0.04	0.00
Si	5.79	0.14
P	0.16	0.01
S	0.10	0.00
K	0.00	0.00
Ca	0.00	0.00
Ti	0.00	0.00
Fe	0.01	0.00
Zr	0.00	0.00
Nb	0.55	0.02
Total:	100.00	

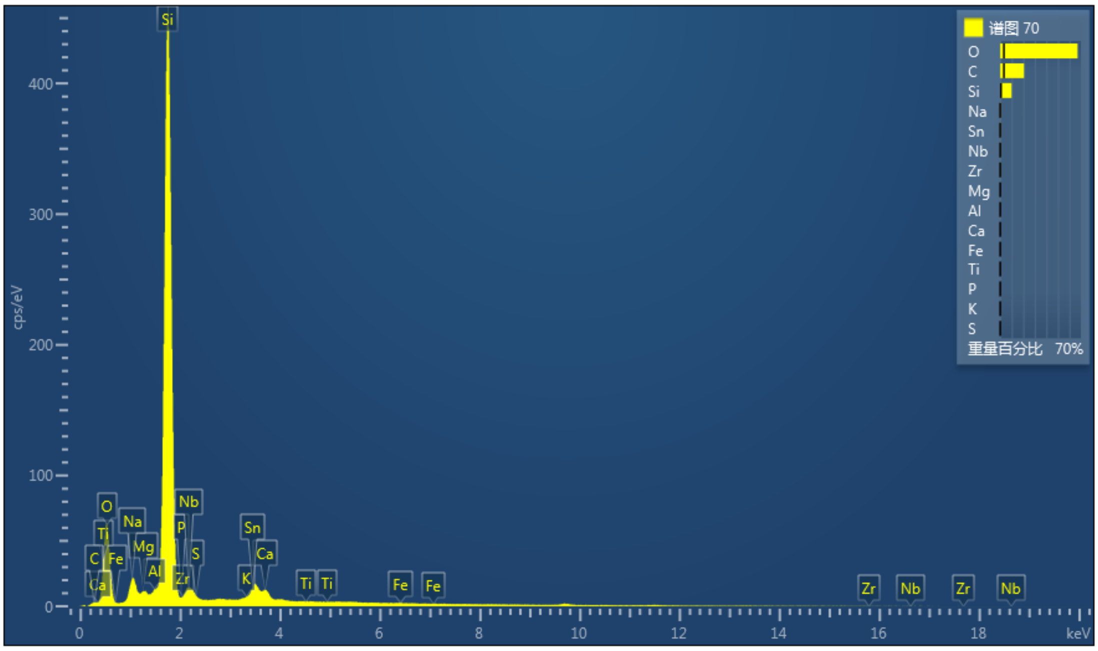

Total	wt%	wt% Sigma
C	20.67	3.25
O	67.16	
Na	0.76	0.06
Mg	0.15	0.01
Al	0.10	0.01
Si	9.80	0.71
P	0.00	0.00
S	0.00	0.00
K	0.00	0.00
Ca	0.07	0.01
Ti	0.00	0.01
Fe	0.01	0.01
Zr	0.24	0.03
Nb	0.46	0.04
Sn	0.56	0.05
Total:	100.00	

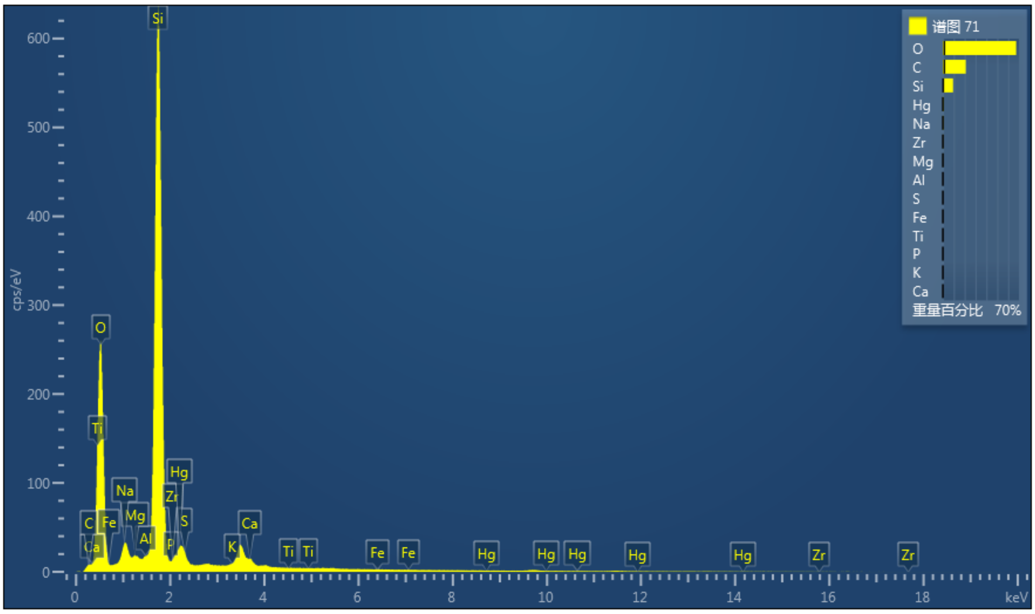

Elements	wt%	wt% Sigma
C	21.10	1.30
O	67.47	
Na	0.68	0.02
Mg	0.13	0.01
Al	0.05	0.01
Si	9.34	0.27
P	0.00	0.00
S	0.04	0.01
K	0.00	0.00
Ca	0.00	0.00
Ti	0.00	0.00
Fe	0.00	0.01
Zr	0.27	0.02
Hg	0.92	0.03
Total:	100.00	

Supplementary Note 6:

EDS report of a striated, ironized cell

Note that the point 54 is the same point shown in Fig. 3i in the main manuscript.

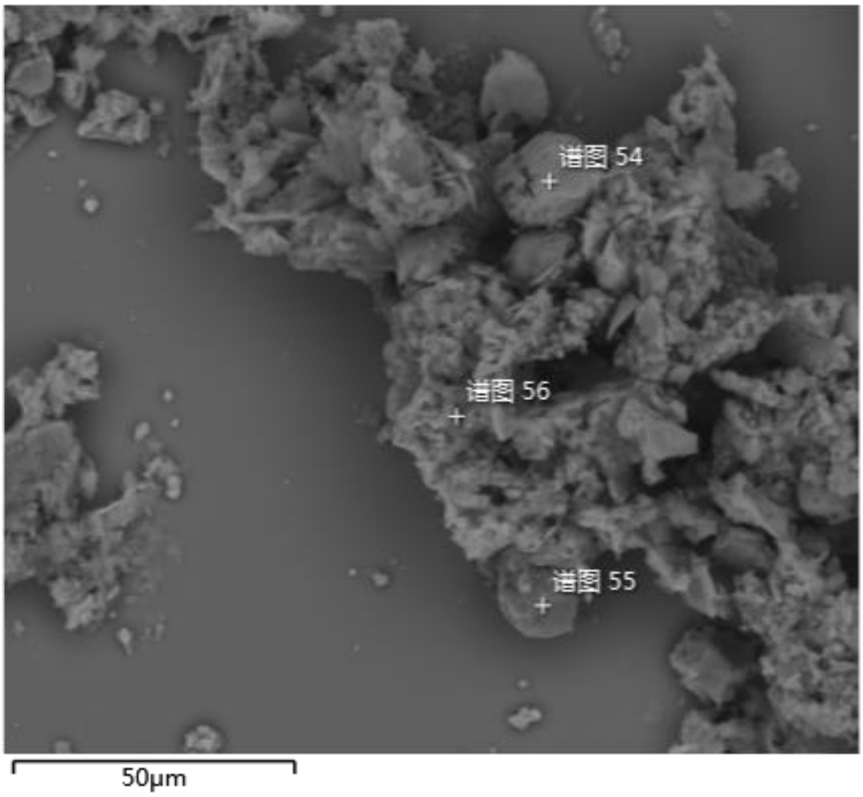

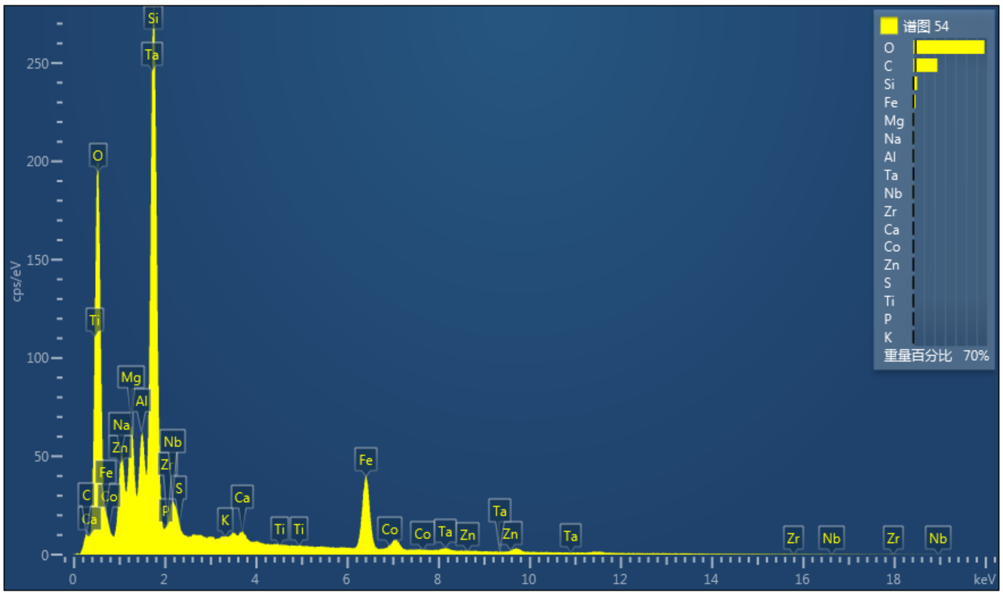

Element	wt%	wt% Sigma
C	22.85	2.10
O	67.56	
Na	0.98	0.05
Mg	1.01	0.05
Al	0.80	0.04
Si	3.46	0.17
P	0.00	0.00
S	0.01	0.01
K	0.00	0.00
Ca	0.06	0.01
Ti	0.01	0.00
Fe	1.98	0.09
Co	0.06	0.01
Zn	0.01	0.01
Zr	0.23	0.02
Nb	0.47	0.03
Ta	0.51	0.11
Total:	100.00	

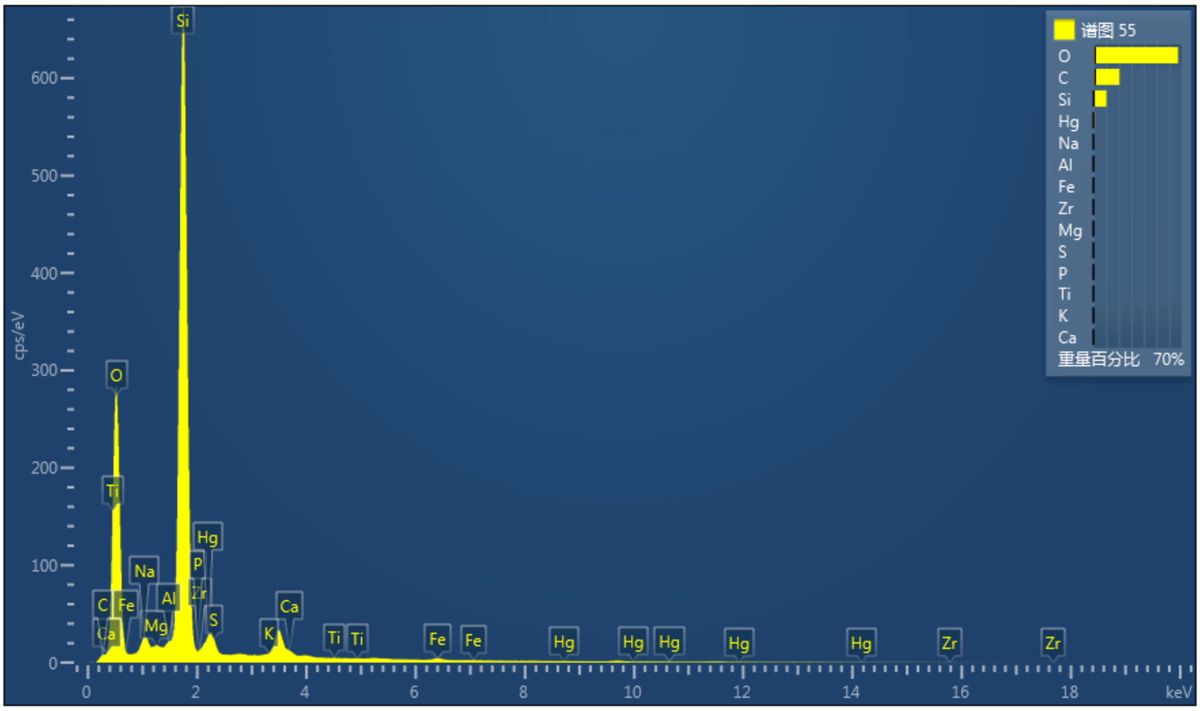

Elements	wt%	wt% Sigma
C	20.60	1.17
O	67.14	
Na	0.48	0.02
Mg	0.10	0.01
Al	0.11	0.01
Si	10.19	0.27
P	0.05	0.01
S	0.06	0.01
K	0.00	0.00
Ca	0.00	0.00
Ti	0.01	0.00
Fe	0.11	0.01
Zr	0.11	0.02
Hg	1.03	0.03
Total:	100.00	

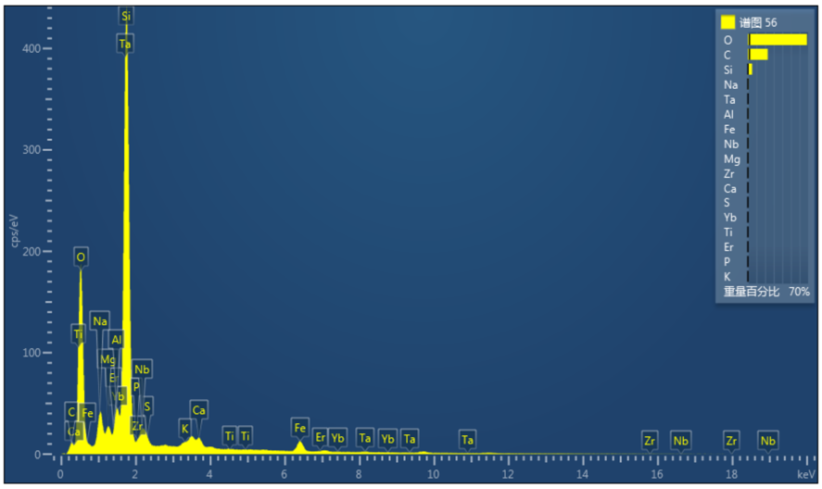

Elements	wt%	wt% Sigma
C	23.09	1.73
O	68.57	
Na	0.76	0.03
Mg	0.22	0.02
Al	0.61	0.04
Si	4.80	0.19
P	0.00	0.00
S	0.04	0.00
K	0.00	0.00
Ca	0.08	0.01
Ti	0.01	0.00
Fe	0.47	0.02
Zr	0.15	0.02
Nb	0.43	0.02
Er	0.00	0.01
Yb	0.02	0.02
Ta	0.74	0.12
Total:	100.00	

Supplementary Note 7:

Supplementary Methods and Tissue Contamination Issues

More detailed protocol for paraffin sections

After demineralization, the extant avian cartilage and the agar-embedded tissues of STM4-3 were subjected to routine dehydration via sequential incubations in 70%, 80%, 90%, 95%, and 100% EtOH for ~ 1 hour each. The tissue blocks were placed in two additional 100% EtOH for 1 hour each to ensure complete dehydration, followed by three 30 min incubations in xylene. Tissues were infiltrated with melted paraffin wax for 30 min each and embedded manually (Paraplast Plus EMS Cat#19216).

Blocks were cooled down in an icy water bath and cut on a microtome (Leica Biosystems RM2265), placed in a warm water bath at 44°C with section adhesive (Tissue-Grip, StatLab), mounted on charged slides (Superfrost Plus, Fisher Scientific), then dried overnight (or a few days) at room temperature.

Paraffin sections were then deparaffinized with xylene (5 min x 2), dehydrated through a graded EtOH series and stained with Hematoxylin and Eosin (see below).

Hematoxylin and Eosin protocol

Solarbio Life Sciences Staining kit- G1120:

<u>Bring sections to H2O:</u>

1. Xylenes: 5 min
2. Xylenes: 5min
3. 100 % EtOh: 5 min
5. 90 % EtOH: 2 min
6. 80% EtOH: 2 min
7. 70% EtOH: 2 min
9. Deinonized water: 2 min
10. Hematoxylin: 15 min (kit says between 5 to 20 min)
11. Rinse well in tap water
12. Differentiation agent: 30 sec
13. Rinse in tap water: 15 min
10. Eosin: 1:30 min (kit says 2 min)
11. Rinse in tap water
12. Rinse in tap water: 2-5 min
15. 95% EtOh : 3s
16. 95% EtOh : 3s
16. 100 % EtOh : 3s
17. 100% EtOh : 1 min

<u>Coverslipping:</u>

Xylenes: 1 min x 4 times

Coverslip with Permount (Fisher Scientific)

Contamination Precautions during paraffin histology and histochemical staining

Dissections and/or fragments extractions were performed in separate rooms and with different designated tools. Demineralization and dehydration steps between the fossil and extant tissues were also performed in two separate rooms, using different equipment at IVPP.

All embedded samples were cut on the same microtome, but not using the same microtome blades between the fossils and the extant blocks of tissues. We did not use the same water for the water-bath (right after the microtome cut) between extant and fossil tissues either. Deparaffinization, dehydration and histochemical staining for the fossil and the extant tissues were made in two different sets of staining dishes and from two different (new and unused) H&E kits. The two sets of solutions and staining dishes did not come in contact with each other at anytime during the staining process. By doing this, we prevented any extant cartilage to 'land' on a slide with fossil cartilage (and *vice versa*).

TEM ultrathin sectioning protocol

The fragment was embedded using the SPI-PON 812 Embedding Kit (MNA, EPOK, DDSA, DMP-30). A mechanical pump vacuum was used during the infiltration process. A stepwise polymerization were applied at 37°C for 12 hours, 45°C for 12 hours, and 60°C for 48 hours [4]. The embedded block was trimmed and cut into ultrathin slices with a thickness of 70 nm using a Leica EM UC7 with a Diamond knife (ultra 45° 3mm). The TEM and STEM images, and the EDS mapping were conducted using a Talos F200X (FEI).

Supplementary Note 8:

How to correctly interpret and use histochemical staining in fossil tissues and discussion on the issue of non-specific staining.

This method must be carefully interpreted since it was originally developed for extant, non-diagenetically altered and non-fossilized tissues. It must imperatively:

- 1) be paired with at least one other complementary method (e.g., a second histochemical stain; and/or ground-sections; and/or SEM for example),
- 2) be carefully interpreted with proper controls (i.e., with extant analogous and/or homologous tissues stained with the same protocols)
- 3) be showing at least two different structures with a morphology and location consistent with structures seen in the extant analogues, as well as the same staining pattern shown by the extant analogues.

Non-specific binding/staining is common in sediments, but if no clear biological structure can be recognized morphologically in the stained material then the staining must be interpreted as non-specific (i.e., meaning that it did not reveal the original biological structures for which the stain was developed for in biological laboratories). For example, in STM4-3, we noticed that beside from the cell with a nucleus (Fig. 4c) all silicified transparent cells stayed transparent after the H&E staining but all brown, ironized cartilage cells (bicc in Fig. 4a) became purple. In this case, either Hematoxylin or Eosin (or both of them) had a chemical affinity for the iron sheath surrounding the brown cartilage cells (this may have to do with its pH or porosity). Since this iron sheath is a non-biological structure (i.e., it was not present around the cartilage cells *in vivo* and was acquired postmortem) and since it stains completely differently from the extant cells (i.e., no extant cell is stained completely purple, they all have an unstained area representing the cytoplasm), the only conclusion to make is a non-specific staining for the brown ironized cells. Every fossil tissue and fossil cell can be carefully interpreted if multiple methods are combined together and if a detailed, high-resolution histological analysis of the stained slides are made, in comparison with extant tissues as controls.

Supplementary References

1 Bailleul, A. M. *et al.* Confirmation of ovarian follicles in an enantiornithine (Aves) from the Jehol biota using soft tissue analyses. *Communications biology* **3**, 1-8 (2020).

2 Liu, X. *et al.* Tissue-like phantoms for quantitative birefringence imaging. *Biomedical optics express* **8**, 4454-4465 (2017).

3 Mayr, G., Kaye, T. G., Pittman, M., Saitta, E. T. & Pott, C. Reanalysis of putative ovarian follicles suggests that Early Cretaceous birds were feeding not breeding. *Scientific Reports* **10**, 1-10 (2020).

4 Luft, J. Improvements in epoxy embedding materials. *J. biophys. biochem. Cytol.* **2**, 799-802 (1961).
